# Diminished preparatory physiological responses in frontotemporal lobar degeneration syndromes

**DOI:** 10.1093/braincomms/fcac075

**Published:** 2022-04-04

**Authors:** Kuan-Hua Chen, Alice Y. Hua, Gianina Toller, Sandy J. Lwi, Marcela C. Otero, Claudia M. Haase, Katherine P. Rankin, Howard J. Rosen, Bruce L. Miller, Robert W. Levenson

**Affiliations:** 1 Department of Psychology, University of California, Berkeley, 2121 Berkeley Way, Berkeley, CA 94720-1650, USA; 2 Memory & Aging Center, Department of Neurology, University of California, San Francisco, San Francisco, CA 94143, USA; 3 Department of Psychiatry and Behavioral Sciences, Stanford University School of Medicine, Stanford, CA 94305, USA; 4 Sierra Pacific Mental Illness, Research, Education and Clinical Centers (MIRECC), Veterans Affairs Palo Alto Health Care System, Palo Alto, CA 94304, USA; 5 Department of Human Development and Social Policy, Northwestern University, Evanston, IL 60208, USA

**Keywords:** lesion, psychophysiology, voxel-based morphometry, resting-state fMRI

## Abstract

Researchers typically study physiological responses either after stimulus onset or when the emotional valence of an upcoming stimulus is revealed. Yet, participants may also respond when they are told that an emotional stimulus is about to be presented even without knowing its valence. Increased physiological responding during this time may reflect a ‘preparation for action’. The generation of such physiological responses may be supported by frontotemporal regions of the brain that are vulnerable to damage in frontotemporal lobar degeneration. We examined preparatory physiological responses and their structural and functional neural correlate in five frontotemporal lobar degeneration clinical subtypes (behavioural variant frontotemporal dementia, *n* = 67; semantic variant primary progressive aphasia, *n* = 35; non-fluent variant primary progressive aphasia, *n* = 30; corticobasal syndrome, *n* = 32; progressive supranuclear palsy, *n* = 30). Comparison groups included patients with Alzheimer’s disease (*n* = 56) and healthy controls (*n* = 35). Preparatory responses were quantified as cardiac interbeat interval decreases (i.e. heart rate increases) from baseline to an ‘instruction period’, during which participants were told to watch the upcoming emotional film but not provided the film’s valence. Patients’ behavioural symptoms (apathy and disinhibition) were also evaluated via a caregiver-reported measure. Compared to healthy controls and Alzheimer’s disease, the frontotemporal lobar degeneration group showed significantly smaller preparatory responses. When comparing each frontotemporal lobar degeneration clinical subtype with healthy controls and Alzheimer’s disease, significant group differences emerged for behavioural variant frontotemporal dementia and progressive supranuclear palsy. Behavioural analyses revealed that frontotemporal lobar degeneration patients showed greater disinhibition and apathy compared to Alzheimer’s disease patients. Further, these group differences in disinhibition (but not apathy) were mediated by patients’ smaller preparatory responses. Voxel-based morphometry and resting-state functional MRI analyses revealed that across patients and healthy controls, smaller preparatory responses were associated with smaller volume and lower functional connectivity in a circuit that included the ventromedial prefrontal cortex and cortical and subcortical regions of the salience network. Diminished preparatory physiological responding in frontotemporal lobar degeneration may reflect a lack of preparation for actions that are appropriate for an upcoming situation, such as approaching or withdrawing from emotional stimuli. The ventromedial prefrontal cortex and salience network are critical for evaluating stimuli, thinking about the future, triggering peripheral physiological responses, and processing and interpreting interoceptive signals. Damage to these circuits in frontotemporal lobar degeneration may impair preparatory responses and help explain often-observed clinical symptoms such as disinhibition in these patients.

## Teaser

Chen *et al.* reported that frontotemporal lobar degeneration syndromes impair patients’ ability to generate increased physiological activity to upcoming emotional stimuli. This effect was associated with functional and structural damage to the patients’ ventromedial prefrontal cortex and salience network. This physiological alteration helps explain patients’ often-observed clinical symptoms such as disinhibition.

## Graphical Abstract

**Graphical abstract:**
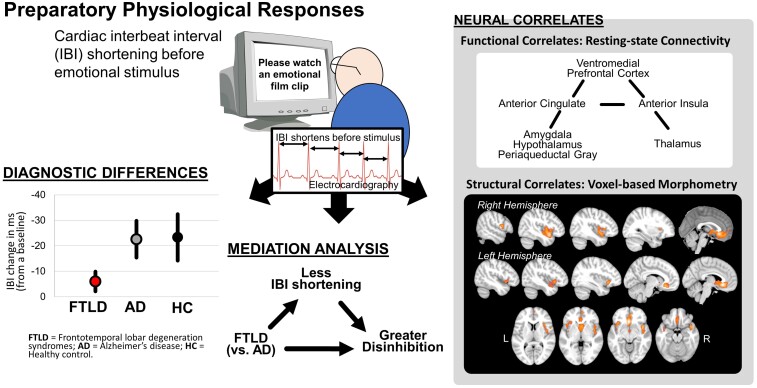

## Introduction

Increased physiological activity often occurs when something significant is about to happen, sometimes even when we have yet to determine the emotional valence of the upcoming stimulus. For example, our heart rate may increase when we are about to unbox a gift or try new food without knowing whether the gift will bring pleasure or the food will taste bad. Such ‘preparatory physiological responses’ (also referred to as ‘preparatory responses’) may reflect a general *preparation for action* that serves to facilitate the subsequent behavioural changes that are tied to emotional responses (e.g. feeling disgusted and displaying withdrawal/expulsion behaviours associated with spoiled food).

### Brain mechanisms for preparatory responses

**Figure 1:**
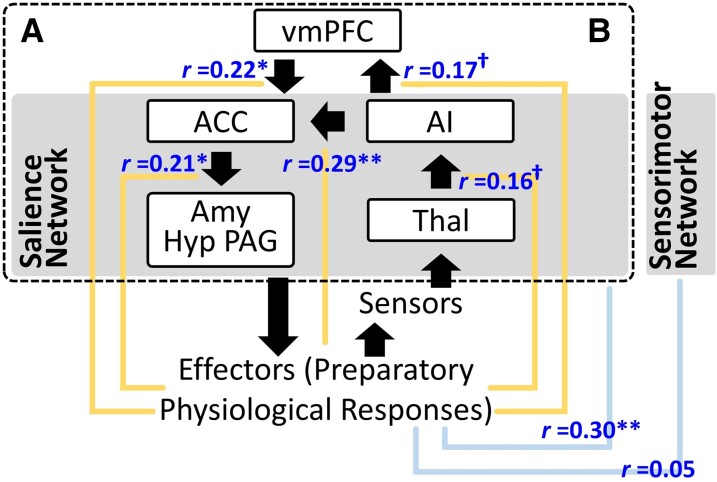
**A brain circuit for preparatory physiological responses.** (**A**) A hypothesized circuit. The dashed black box represents the entire circuit. The five solid black boxes represent cortical and subcortical regions involved in this process. The back arrows represent signal flows. **(B)** Functional connectivity results. The blue font by the yellow lines indicates the correlation coefficients between each node-to-node connectivity (e.g. AI–ACC, AI–vmPFC) and preparatory physiological responses; the blue line indicates the correlation coefficient between preparatory physiological responses and the vmPFC–SN circuit’s overall functional connectivity. Note that prior to data analyses, connectivity between the three subcortical efferent regions (i.e. Amy, Hyp, PAG) and ACC were averaged together. In addition, connectivity between and within each hemisphere were also averaged for each pair of brain regions (nodes) of interests. ^Ϯ^*P* < 0.10; *P < 0.05; **P < 0.01; ***P < 0.001.

Although the precise brain mechanisms underlying these preparatory responses remain undetermined, findings from previous research suggest that multiple brain regions and networks may play critical roles in this process (Fig. 1A). The ventromedial prefrontal cortex (vmPFC) may be involved in evaluating situations and generating predictions (e.g. based on previous experiences, food could be either rewarding or punishing).^1,2^ The vmPFC can also communicate with the salience network (SN)^3^—including cortical areas such as the posterior region of the anterior cingulate cortex (ACC) and subcortical areas such as the amygdala (Amy), hypothalamus (Hyp) and periaqueductal gray (PAG)—resulting in adjustments in the autonomic and somatic nervous systems (e.g. increased heart rate to support possible approach or avoidance behaviours).^4^ The anterior insula (AI) and thalamus (Thal) in the SN may also be involved by providing the ACC and vmPFC with information about current bodily states (e.g. levels of cardiovascular activity, muscle contraction/relaxation) through proprioceptive and interoceptive feedback.

### Preparatory responses in FTLD

Frontotemporal lobar degeneration syndromes (FTLDs) provide an ideal model for studying the preparatory responses. FTLD consists of a group of clinically, genetically, and pathologically related clinical disorders, including behavioural variant frontotemporal dementia (bvFTD), semantic variant primary progressive aphasia (svPPA), non-fluent variant primary progressive aphasia (nfvPPA), corticobasal syndrome (CBS) and progressive supranuclear palsy (PSP). In FTLD, neurodegeneration commonly occurs in frontal and anterior temporal brain regions,^5^ which overlap with the aforementioned brain regions that may be involved in generating preparatory responses. Patients with FTLD also commonly develop behavioural symptoms such as apathy and impulsivity/disinhibition,^6,7^ which could reflect altered preparatory responses that then contribute to inappropriate subsequent behaviours (or lack thereof). For instance, in the example above, patients may have difficulty activating avoidance behaviours to contaminated food because their heart rate has not increased enough to support the somatic adjustments needed for such behaviours.

Although preparatory responses have not been studied in FTLD (nor in healthy adults), numerous studies have demonstrated diminished physiological responses in patients with bvFTD either after stimulus onset or when the emotional valence of an upcoming stimulus are revealed. Compared to healthy controls (HCs), patients with bvFTD have shown diminished physiological responses to disgust-eliciting films^8^ and unpleasant smells.^9^ Orienting responses to emotional stimuli,^10–12^ which are typically characterized by decreased heart rate, are also diminished in bvFTD.^13^ Patients with bvFTD also showed smaller skin conductance responses when they were told that an unpleasant smell would be delivered in 15 s. Importantly, these impairments have been associated with structural degeneration in the vmPFC and SN.^14–16^

### The present study

The present study examines preparatory responses in FTLD. We quantified preparatory responses as decreases in cardiac inter-beat intervals (IBIs; or increases in heart rate) from a pre-trial *baseline* period to an *instruction* period when participants were told that they would be watching a film clip but had not yet been provided information about the emotional valence. We focused on changes in IBI because they serve an essential role in providing metabolic support for somatic motor activities^17^ that are important for subsequent coping behaviours. Changes in IBI also happen more rapidly than changes in other physiological measures (e.g. electrodermal responses and skin temperature),^18^ allowing us to observe preparatory responses that could be very transient before the stimulus onset. We also quantified orienting responses as IBI changes from the *instruction* period to the first 6 s of the *film* clip. This enabled us to determine whether these two different responses were similarly affected across diagnostic groups and whether they are associated with different neural correlates. To determine whether diminished preparatory responses helped explain often-observed clinical symptoms in FTLD, patients’ behavioural symptoms of apathy and disinhibition^6,7^ were assessed using the neuropsychiatric inventory (NPI).^19^

We made three hypotheses. First, because FTLD targets frontal and temporal regions of the brain (particularly the vmPFC and SN in bvFTD),^5^ we hypothesized that FTLD as a group would have impaired preparatory responses compared to HC and Alzheimer’s disease, which is characterized by different patterns of neurodegeneration and clinical symptoms,^20,21^ and that this impairment would be strongest in bvFTD. Second, we hypothesized that FTLD would exhibit greater behavioural symptoms than Alzheimer’s disease^6,7^ and that these group differences would be mediated by greater impairments in preparatory responses in FTLD. Third, consistent with the neural circuitry described above (Fig. 1A), we hypothesized that greater impairment in preparatory responses would be associated with smaller gray matter volume and lower resting functional connectivity within the vmPFC and regions of the SN (e.g. vmPFC–ACC, AI–ACC).

## Materials and methods

### Participants

Participants included 276 patients (76 bvFTD, 38 svPPA, 31 nfvPPA, 36 CBS, 33 PSP and 62 Alzheimer’s disease) and 38 HCs. All patients were recruited from the Memory and Aging Centre (MAC) at the University of California, San Francisco (UCSF) between 2006 and 2016 in a collaborative research project between the MAC and the Berkeley Psychophysiology Laboratory at the University of California, Berkeley (UCB). At UCSF, patient diagnoses were determined by a multidisciplinary team that consisted of neurologists, nurses, clinical psychologists and neuroscientists (by reviewing clinical interviews and patients’ neurological, neuropsychological, neuroimaging testing data) using current research criteria for bvFTD,^22^ svPPA, nfvPPA,^23^ CBS,^24^ PSP^25,26^ and Alzheimer’s disease.^20^ HCs without a history of neurological or psychiatric disorders were recruited from the community via advertisements.

### Procedure

All participants first visited UCSF, where they underwent detailed clinical interviews (with their caregivers), neurological examination, functional assessment, neuropsychological evaluation, structural MRI and resting-state functional MRI (rs-fMRI). Following this UCSF visit (4 months for patients and 12 months for HCs), participants visited UCB for a comprehensive assessment of emotional functioning.^27^ Informed consent was obtained upon arrival at both sites. Procedures were approved by the UCSF and UCB Institutional Review Boards.

**Figure 2:**
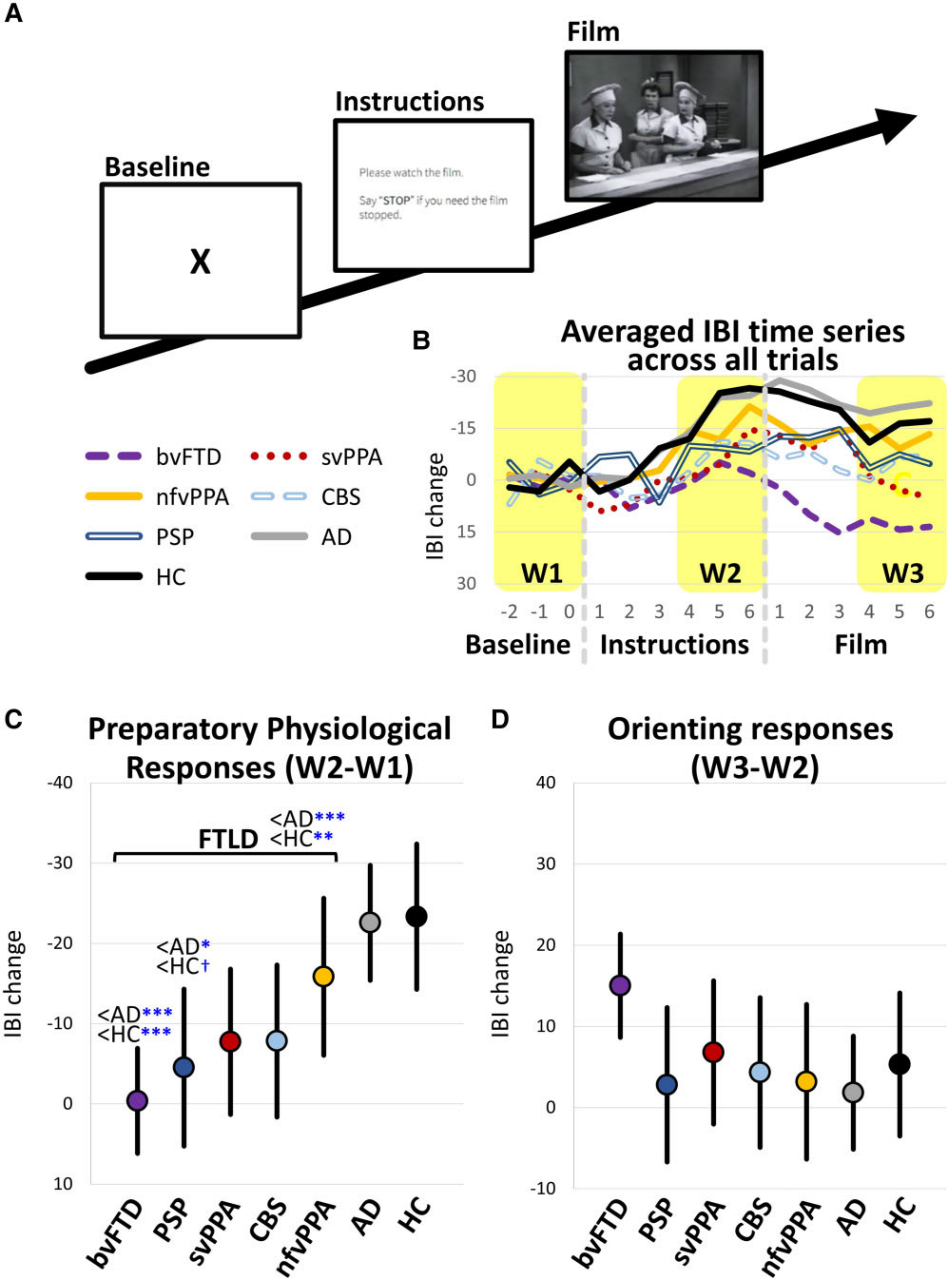
**Task procedures and the quantification of preparatory and orienting physiological responses across diagnostic groups.** (**A**) Task procedure. The film watching task consisted of three trials. In each trial, participants sat for a 60 s baseline period and then were presented with instructions for 6 s, which informed them the film was about to start. Immediately following the instructions, participants watched a film clip that lasted between 87 and 106 s. **(B)** Averaged time series of cardiac IBIs across all three film trials for the seven diagnostic groups. Preparatory physiological responses were quantified as IBI change from the last 3 s of the rest period to the last 3 s of the instruction period (i.e. periods B-A). Orienting responses were quantified as IBI changes from the last 3 s of the instruction period to the second 3 s of the film period (i.e. period C-B). **(C)-(D)** Averaged preparatory and orienting responses by diagnostic group, *Mean* ± 95% confidence intervals. Annotations indicate significant or trending effects as compared to the two comparison groups (i.e. Alzheimer’s disease and HC) revealed by ANOVA and *post hoc* comparisons. ^Ϯ^*P* < 0.10; *P < 0.05; **P < 0.01; ***P < 0.001.

The present study focused on a film-viewing task, which was the first task in the UCB assessment. Before the task, non-invasive physiological sensors were applied to the participants. The task consisted of three trials. Participants were informed that they would be watching several short films. Each trial began with a 60 s *baseline period* that started with participants being asked to watch an ‘X’ on the centre of the screen (Fig. 2A). Next, there was a six-second *instruction period* during which the screen displayed: ‘Please watch the film. Say stop if you need the film stopped.’ After the instruction period, there was a *film period* (86–106 s) in which participants watched a film selected to induce amusement (trial 1), sadness (trial 2) and disgust (trial 3).^8,28,29^ The order of the films was fixed across participants. For additional details about the procedure, see Supplementary Procedure.

Later in the UCB assessment, participants also completed an acoustic startle task, where they sat for a 60 s baseline and heard a brief (100-ms) and loud (115-dB) burst of white noise without warning. Our previous research^30^ has demonstrated that this task produces marked physiological responses in HCs and patients with FTLD and Alzheimer’s disease. In the present study, IBI change in response to this simple, loud sensory stimulus was included as a covariate to adjust for individual differences in *overall physiological responding*.

### Physiological measures

#### Data acquisition and processing

Physiological data including electrocardiogram (ECG) and other physiological measures (e.g. electrodermal, somatic, respiration; data not presented here) were obtained using a BIOPAC MP150 system. For ECG, Beckman miniature electrodes with Redux paste were placed on opposite sides of the participant’s chest, which were connected to a BIOPAC ECG10C amplifier, and a computer with analog-to-digital capability that sampled the signal at 300 Hz. Using a programme written by R.W.L., IBI was calculated as the interval between successive R-waves and then averaged every second. Trained research assistants examined the second-by-second data to identify and remove artefacts. Among the 314 participants enrolled in this study, 29 were excluded from analyses due to poor data quality (e.g. excessive movement artefact; Supplementary Physiological Methods describes details about data exclusion). The remaining 285 participants included 67 bvFTD, 35 svPPA, 30 nfvPPA, 32 CBS, 30 PSP, 56 Alzheimer’s disease and 35 HC. Table 1 shows their sociodemographic and functional characteristics.

**Table 1:** Sociodemographic and functional characteristics of participants in main data analyses (*n* = 285)

		FTLD syndromes								
	Total sample	bvFTD	svPPA	nfvPPA	PSP	CBS	Alzheimer’s disease	HC	*F/X^2^*	*P*
***n***	**285**	67	35	30	30	32	56	35		
**Gender**									14.91	0.02
Men	**150**	46	21	13	17	15	26	12		
Women	**135**	21	14	17	13	17	30	23		
**Handedness**									9.22	0.16
Right	**241**	60	34	26	22	29	46	24		
Left/ambidextrous	**32**	6	1	4	7	3	9	2		
N/A	**12**	1	0	0	1	0	1	9		
**Race**									23.94	0.77
White/European American	**254**	60	31	26	24	29	52	32		
Black/African-American	**2**	0	0	0	0	1	1	0		
Latinx/Chicanx American	**10**	1	1	2	3	0	2	1		
Asian American	**16**	5	2	2	2	2	1	2		
Multi-racial/prefer to self-describe	**2**	0	1	0	1	0	0	0		
N/A	**1**	1	0	0	0	0	0	0		
**Age**	**64.69 (7.82)**	62.19 (8.14)*	63.85 (5.85)	68.61 (7.02)	67.34 (6.75)	66.10 (5.61)	62.55 (8.75)*	66.85 (8.24)	4.69	<0.001
**Education**	**16.54 (3.10)**	16.12 (3.10)	16.60 (2.76)	16.47 (3.83)	17.41 (3.45)	16.26 (3.64)	16.38 (2.77)	17.21 (2.11)	0.88	0.51
**Dementia severity (CDR-Total)**	**0.73 (0.56)**	1.157 (0.62)***	0.66 (0.42)***	0.48 (0.43)***	0.87 (0.39)***	0.63 (0.46)***	0.83 (0.38)***	0 (0)	28.75	<0.001
**Dementia severity (CDR-Box)**	**4.04 (3.14)**	6.49 (3.18)***	3.87 (2.43)***	1.88 (1.94)**	5.60 (2.56)***	3.47 (2.39)***	4.40 (2.16)***	0 (0)	34.87	<0.001
**Cognitive functioning (MMSE)**	**23.93 (6.27)**	23.76 (6.87)***	24.14 (4.89)**	24.35 (6.06)**	25.60 (3.84)	23.27 (7.16)***	20.66 (6.43)***	29.64 (0.57)	7.30	<0.001
**Overall physiological responding** **(IBI changes in response to a loud white noise)**	**−44.41 (72.63)**	**−**55.85 (88.53)	**−**51.75 (62.30)	**−**35.73 (60.39)	**−**17.90 (67.76)	**−**49.37 (69.12)	**−**30.91 (65.80)	**−**66.51 (72.62)	1.85	0.09

Mean (SD). F/X^2^ = main effects of diagnostic groups revealed by one-way ANOVAs or chi-squared tests. Annotations indicate significant or trending effects (*post hoc*) as compared to the HC group. *P < 0.05; **P < 0.01; ***P < 0.001.

#### Preparatory physiological responses

Preparatory responses were quantified as the *change* in the averaged IBI of the last three seconds of the baseline period and seconds 4–6 of the instruction period (Fig. 2B; time windows W2–W1). Decreased IBI values correspond to increased heart rate, representing greater preparatory responses. The first three seconds of the instruction period were not included because preparatory responses may still be building at this time (Fig. 2B). In preliminary analyses, a repeated-measures analysis of variance (ANOVA) (3 trials × 7 diagnostic groups) did not reveal any significant effects for trial order [*F*(2, 556) = 0.90, *P* = 0.41] or trial × diagnostic group interactions [*F*(12, 556) = 1.26, *P* = 0.24]. Therefore, responses from all trials were averaged.

#### Orienting responses

Preliminary analyses revealed an IBI increase in comparison groups (i.e. Alzheimer’s disease and HC) that generally started at the onset of the film and peaked approximately four to six seconds after film onset. Therefore, orienting responses were quantified as *changes* in average IBI from the last 3 s of the instruction period to seconds 4–6 of the film period (Fig. 2B; time windows W3–W2). Increased IBI values correspond to decreased heart rate, representing greater orienting responses. Like preparatory responses, the first 3 s of the film were not included in the analyses because orienting responses were still building during this time. Responses from all trials were averaged.

### Functional measures

#### Dementia severity

The Clinical Dementia Rating (CDR)^31^ Scale assessed dementia severity. CDR total score (CDR-Total; 0 = normal, 0.5 = very mild dementia; 1 = mild dementia, 2 = moderate dementia, 3 = severe dementia) and the sum of boxes score (CDR-Box; range: 0 to 18, with higher values indicating greater severity) were used. Using the same approach as previous studies,^32,33^ CDR-Total was used to determine participants’ eligibility to be included in functional connectivity analyses (i.e. participants with CDR >1 were ineligible due to severe loss of brain tissue). CDR-Box was added as a covariate because higher scores typically correlate with greater severity of neurodegeneration.^34^

#### Cognitive functioning

The Mini-Mental State Exam (MMSE)^35^ was used to assess global cognitive functioning. MMSE scores range from 0 to 30, with higher values indicating greater cognitive functioning. Scores were added as a covariate to ensure our findings did not simply reflect patients’ cognitive impairment.

#### Overall physiological responding

Overall physiological responding was quantified as *changes* in average IBI from the last three seconds of a baseline period to the first 6 s after the presentation of the loud noise in the acoustic startle task. We chose this 6 s time window because our preliminary analysis revealed an overall IBI decrease (or heart rate increase) during this period (Table 1 and Supplementary Fig. 1). IBI change scores were inverted so higher values corresponded to larger responses to the loud noise. We used this as a covariate to ensure any preparatory response findings did not simply reflect changes in overall physiological responding.

#### Apathy and disinhibition

The NPI was administered by conducting a semi-structured interview with each patient’s caregiver. The NPI included 12 neuropsychiatric symptoms that are frequently seen in neurodegenerative disorders.^7,19^ The present study focused on apathy and disinhibition—the two symptoms that are the most prominent in FTLD.^6,7^ Higher scores reflected more frequent or severe symptoms.

### Neuroimaging measures

#### Data acquisition and preprocessing

We obtained structural MRI data using 1.5 T (*n* = 9), 3 T (*n* = 176) or 4 T (*n* = 37) research-quality scanners for 222 participants (43 bvFTD, 30 svPPA, 27 nfvPPA, 31 CBS, 28 PSP, 43 Alzheimer’s disease and 20 HC). MRIs were visually inspected for scan quality (e.g. no motion or metal artefact). We utilized statistical parametric mapping version 12 (SPM12) default parameters for preprocessing structural MRI data (for details, see Supplementary Neuroimaging Methods). We also characterized the areas of neurodegeneration for each patient group by examining structural differences in gray matter maps between each patient group and HC. These results are presented in Supplementary Fig. 2.

Task-free functional MRI images were also obtained in a subsample of 117 participants (17 bvFTD, 14 svPPA, 19 nfvPPA, 17 PSP, 22 CBS, 20 Alzheimer’s disease and 8 HC) who were scanned on the 3 T scanner. Participants were instructed to relax with their eyes closed for 8 min. rs-fMRI data were preprocessed using SPM12. Node-pair intrinsic connectivity analysis^36,37^ was applied to identify the functional connectivity between our hypothesized brain regions that support preparatory responses. Within each participant, pairwise correlation coefficients were calculated between a set of cortical and subcortical regions-of-interest (ROIs), including the vmPFC, ACC, Amy, Hyp, PAG, Thal and AI. MARSBAR was used to create spherical ROIs centred on Montreal Neurological Institute (MNI) coordinates based on previous studies.^38–40^Supplementary Neuroimaging Methods describes parameters for data preprocessing including MNI coordinates for ROIs. To test our hypothesized neural circuit, we calculated regional summary scores by averaging each participant’s correlation coefficients (within and between hemispheres) within the following pairs of nodes: (i) vmPFC and ACC, (ii) ACC and all subcortical regions combined—including the Amy, Hyp and PAG, (iii) Thal and AI, and (iv) AI and ACC and (v) AI and vmPFC. For each participant, we averaged these five correlation coefficients to obtain an *overall index* of functional connectivity for our hypothesized circuit.

### Statistical analysis

To test Hypothesis 1, we performed a one-way ANOVA to determine diagnostic group differences in preparatory responses. To compare, we performed the same analysis for orienting responses. To ensure our findings were robust, we repeated these analyses using ANCOVAs and included covariates that significantly differed between diagnostic groups (i.e. age, gender, dementia severity [CDR-Box], and cognitive functioning [MMSE]; Table 1). Significant group effects were followed by two-tailed *post hoc* comparisons using the *Bonferroni* method to correct for multiple comparisons.

To test Hypothesis 2, we first performed bivariate correlations (two-tailed) to evaluate the associations between preparatory/orienting responses and apathy and disinhibition scores. We next performed independent-sample *t*-tests to determine whether the previously reported group differences in apathy and disinhibition between FTLD and AD^6,7^ would be observed in our sample. We then conducted two mediation analyses (using SPSS PROCESS 3.4.1 default parameters)^41^ to test whether group differences (FTLD = 1 versus Alzheimer’s disease = 0) in disinhibition and/or apathy were mediated by levels of preparatory/orienting responses. To ensure findings were robust, we repeated these analyses and included overall physiological responding (i.e. IBI changes in response to the acoustic startle stimulus), which significantly correlated with preparatory responses (Supplementary Table 1).

To test Hypothesis 3, whole-brain voxel-based morphometry (VBM) analyses were performed, using a multivariate linear regression to examine areas of smaller volume associated with smaller preparatory/orienting responses. We examined statistical maps and reported findings at *P*_FWE_ < 0.05. The minimum cluster size reported was 350 mm3. We ran 5000 permutation analyses to derive a study-specific error distribution^42^ using vlsm2^43^ (see Supplementary Neuroimaging Data Analysis for more details). Analyses were adjusted for six diagnostic dummy variables (1 = patient diagnosis of interest; 0 = remaining groups) to ensure that our findings did not simply reflect diagnostic differences, two dummy variables for three different scanner types, total intracranial volume (TIV; to account for head size) and two functional covariates that significantly correlated with preparatory responses (i.e. dementia severity and overall physiological responding; Supplementary Table 1). For functional connectivity analyses, bivariate correlations and linear regressions were performed to examine the associations between preparatory/orienting responses and overall and node-pair connectivity. All analyses were adjusted for six diagnostic dummy variables and two covariates that significantly correlated with preparatory responses (i.e. age and overall physiological responding; Supplementary Table 1). We also performed analyses without adjusting for these covariates and present these results in Supplementary Table 2.

For all analyses, effects with *P* < 0.05 were considered statistically significant.

### Data availability

Study data are available upon request from the corresponding author. The data are not publicly available due to their containing information that could compromise the privacy of research participants.

## Results

### Diagnostic group differences

#### Preparatory physiological response

When comparing FTLD (all syndromes combined), Alzheimer’s disease and HC, an ANOVA revealed a group effect, *F*(2, 282) 11.80, *P* < 0.001, Fig. 2C. Pair-wise *post hoc* comparisons indicated that the FTLD group had smaller preparatory responses (i.e. less pronounced IBI decreases or heart rate increases) than Alzheimer’s disease (*P* < 0.001) and HC (*P* = 0.002). No significant group differences emerged between HC and Alzheimer’s disease. When comparing each of the five FTLD syndromes to HC and Alzheimer’s disease, an ANOVA revealed syndrome group effects, *F*(6, 278) = 5.17, *P* < 0.001. *Post hoc* comparisons between each FTLD syndrome and Alzheimer’s disease or HC (total *n* of comparisons = 10; *Bonferonni* corrected) indicated smaller preparatory responses in bvFTD than in Alzheimer’s disease and HC (*P*s *<*0.001). PSP also had smaller responses than Alzheimer’s disease (*P* = 0.036). No other statistically significant comparisons emerged between FTLD syndromes and comparison groups.

Additional analyses were performed to examine the robustness of the findings above. To ensure these effects were not driven by demographic or functional differences between diagnostic groups, we repeated the analyses above with variables that significantly differed between groups as covariates. To ensure our findings from analyzing averaged IBI during seconds 4–6 of the instruction period were robust, we repeated our analyses using averaged IBI during the entire six seconds of the instruction period. To ensure our findings were not biased by increased knowledge about the task after the first trial, we repeated the above ANOVAs while replacing the averaged preparatory responses across all three trials with preparatory responses from only the first trial. To ensure our effects were not driven by participants’ incorrect belief that the films would all be negatively valenced, we analysed preparatory responses in the second trial only, which took place after participants watched the first trial’s amusement film (and thus realized the films could also be positive). These tests of robustness supported the group effects reported above (*P*s <0.05; Supplementary Figs. 3 and 4).

#### Orienting response

ANOVAs did not reveal any group differences between FTLD, Alzheimer’s disease and HC, *F*(2, 272) _=_ 1.22, *P* = 0.30, or between each FTLD syndrome, Alzheimer’s disease and HC, *F*(6, 278) = 1.67, *P* = 0.13, Fig. 2D.

### Mediation effects

#### Preparatory physiological response

**Figure 3:**
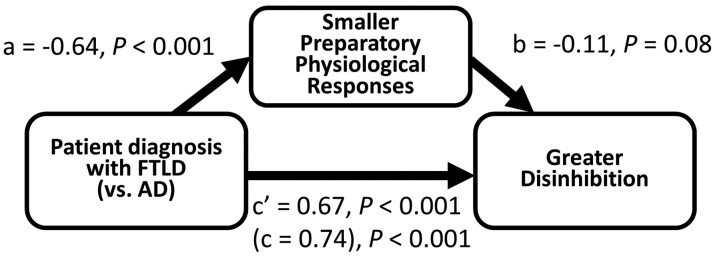
**Results of mediation analysis.** Preparatory physiological responses as a mediator for the effects of greater disinhibition in FTLD (versus Alzheimer’s disease). Standardized indirect effect = 0.07, 95% CI [0.0030, 0.1591], accounting for 9.11% of the total effect.

Prior to data analyses, we inverted preparatory response scores, so that higher values corresponded to greater responses. Correlation analyses revealed an association between lower preparatory responses and greater disinhibition (*r* = −0.18, *P* = 0.004). A *t*-test revealed FTLD displayed more disinhibition than Alzheimer’s disease, *t*(1, 239) = 4.92, *P* < 0.001, Cohen’s *d* = 0.85. Mediation analyses revealed that this group difference was mediated by lower preparatory responses (standardized indirect effect = 0.07, 95% CI [0.0030, 0.1591], accounting for 9.11% of the total effect) (Fig. 3). The mediation effect remained marginally significant when the analyses adjusted for overall physiological responding (standardized indirect effect = 0.06, 90% CI [0.0010, 0.1331], accounting for 8.15% of the total effect, Supplementary Fig. 5).

Correlation analyses also revealed an association between lower preparatory responses and greater apathy (*r* = -0.13, *P* = 0.048). In addition, apathy was also greater in FTLD than Alzheimer’s disease, *t*(1, 239) = 2.92, *P* = 0.004, Cohen's *d* = 0.47. However, this group difference was not significantly mediated by preparatory responses (Supplementary Fig. 6).

#### Orienting response

Larger orienting responses were correlated with greater apathy (*r* = 0.18, *P* = 0.004) but not disinhibition (*r* = 0.09, *P* = 0.18). No mediation effects emerged for orienting responses (Supplementary Fig. 7).

### Structural neural correlates

#### Preparatory physiological responses

**Figure 4:**
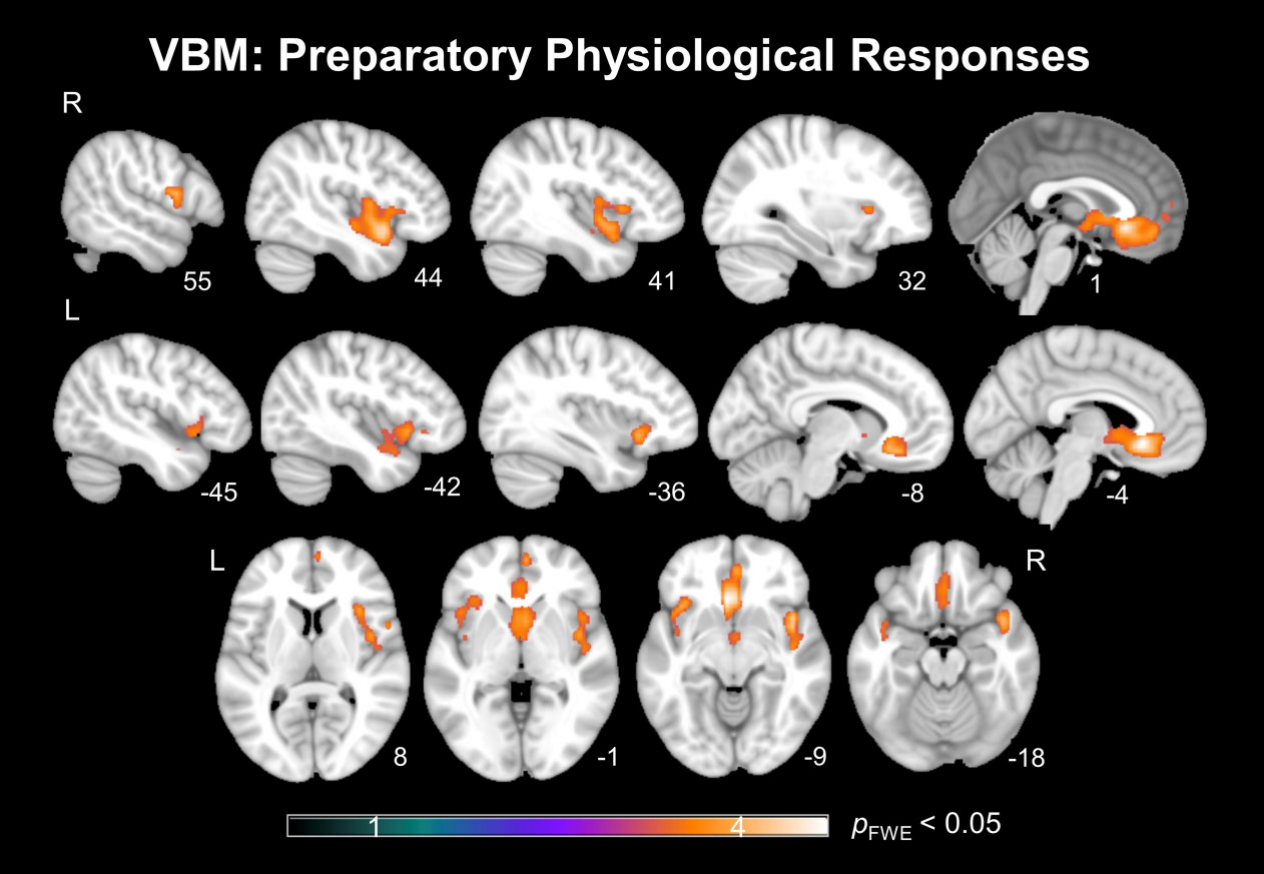
**Results of full-brain voxel-based morphometry analyses.** *T*-score map of brain areas for which smaller gray matter volume was associated with smaller preparatory physiological responses after adjusting for diagnostic group, scanner type (two scanner type variables), TIV, overall physiological functioning and disease severity (CDR-Box). Three large clusters emerged in the (**A**) bilateral vmPFC and caudate; (**B**) right AI, right superior temporal pole, right Rolandic operculum and right Heschl’s gyrus; and (**C**) in the left ventral AI, left orbitofrontal frontal gyrus, and left superior temporal pole (*P*_FWE_ < 0.05).

A whole-brain VBM analysis revealed that smaller preparatory responses were associated with smaller gray matter volume in three large clusters (Table 2, Fig. 4): vmPFC, extending to the anterior ACC and bilateral caudate; right AI, extending to the right superior temporal pole, right rolandic operculum and Heschl’s gyrus; and left ventral AI, extending to the left inferior orbital frontal gyrus and left superior temporal pole.

**Table 2:** Structural neural correlates of preparatory physiological responses

Anatomical region	Volume mm^3^	*x*	*y*	*z*	Max *T*	Corrected *P*
Left vmPFC	10 969	**−**4	24	**−**9	4.67	0.0056
Right vmPFC	a					
Bilateral caudate	a					
Bilateral anterior ACC	a					
Right AI	7607	44	10	**−**12	4.31	0.0108
Right superior temporal pole	a					
Right rolandic operculum	a					
Right Heschl’s gyrus	a					
Left ventral AI	3213	**−**36	20	**−**8	3.88	0.0284
Left inferior orbital frontal gyrus	a					
Left superior temporal pole	a					

Analyses adjusting for six diagnostic variables, scanner type, TIV, overall physiological responding (IBI change in response to a loud white noise) and disease severity (CDR-Box). Results considered significant at *P*_FWE_ < 0.05. ^a^Signifies that these regions were included in the cluster above.

#### Orienting response

No neural correlates emerged for orienting responses.

### Functional connectivity neural correlates

#### Preparatory physiological responses

Correlation analyses revealed that smaller preparatory responses were associated with weaker connectivity between (i) the vmPFC and ACC (*r* = 0.22, *P* = 0.022); (ii) ACC and subcortical SN regions (*r* = 0.21, *P* = 0.032); and (iii) AI and ACC (*r* = 0.29, *P* = 0.002). We also observed an association between smaller preparatory responses and weaker overall connectivity within the vmPFC-SN circuit (*r* = 0.30, *P* = 0.001; Fig. 1B and Supplementary Table 2).

To ensure these findings were specific to our hypothesized vmPFC-SN circuit, we included a ‘control’ brain network—the sensorimotor network (SMN; Supplementary Neuroimaging Data Preprocessing describes methods for computing SMN’s overall connectivity). A correlation analysis did not reveal a relationship between preparatory responses and SMN connectivity (*r* = 0.05, *P* = 0.576; also see Supplementary Table 2). A linear regression including both the vmPFC-SN and SMN overall connectivity in the same model revealed that only the vmPFC-SN’s connectivity predicted levels of preparatory responses (*β* = 0.33, *P* = 0.001; Table 3, model 1).

**Table 3:** Functional connectivity (linear regression model 1: overall connectivity; model 2: node-pair connectivity) correlates of preparatory physiological responses

	Preparatory Physiological responses				Orienting responses			
	Model 1		Model 2		Model 1		Model 2	
	*Beta*	*P*	*Beta*	*P*	*Beta*	*P*	*Beta*	*P*
**Diagnostic covariates**								
bvFTD	−0.02	0.906	−0.01	0.961	0.30	0.061	0.31	0.052
svPPA	−0.04	0.791	−0.01	0.926	0.06	0.667	0.06	0.717
nfvPPA	0.09	0.553	0.11	0.460	0.07	0.658	0.06	0.732
CBS	0.04	0.818	0.05	0.771	0.02	0.889	−0.01	0.945
PSP	0.03	0.816	0.04	0.793	0.00	0.980	−0.03	0.842
Alzheimer’s disease	0.17	0.255	0.18	0.231	0.00	0.999	−0.02	0.893
**Demographic and functional covariates**								
Age	−**0**.**18**	**0**.**048**	−**0**.**20**	**0**.**034**	−0.14	0.150	−0.13	0.200
Overall physiological responding	**0**.**29**	**0**.**001**	**0**.**31**	**0**.**001**	−0.12	0.201	−0.13	0.195
**Functional networks**								
SMN	−0.09	0.368	−0.14	0.195	−0.09	0.400	−0.07	0.541
vmPFC–SN	**0**.**33**	**0**.**001**			0.06	0.610		
vmPFC–ACC			0.15	0.152			0.00	0.979
ACC–Amy/Hyp/PAG			0.08	0.454			0.04	0.726
Thal–AI			−0.04	0.713			0.05	0.674
AI–ACC			**0**.**23**	**0**.**043**			0.06	0.659
AI–vmPFC			0.06	0.594			−0.10	0.401

For preparatory physiological responses, higher values indicate larger responses (i.e. greater IBI decrease). For functional networks, analyses included our hypothesized vmPFC-SN circuit and a control SMN network. Italic font indicates node–pair connectivity within the vmPFC-SN network. Bolded font indicates significant effects at the threshold of *P* < 0.05.

Next, within our hypothesized vmPFC-SN circuit, we determined which pair(s) of node-to-node connectivity were specifically critical for preparatory responses. When the SMN overall connectivity was adjusted, a linear regression with all five node-pairs entered (Table 3, model 2) revealed an association between lower AI–ACC connectivity and smaller preparatory responses (*β* = 0.23, *P* = 0.043). No other significant effects were found.

#### Orienting response

We performed the same correlation and linear regression analyses for orienting responses but did not find any significant effects (Supplementary Table 2 and Table 3).

## Discussion

We found that FTLD, specifically bvFTD, exhibited smaller preparatory responses than Alzheimer’s disease and HCs. We also observed similar but somewhat weaker effects for PSP. No group differences were found for CBS, svPPA or nfvPPA when compared to Alzheimer’s disease and HCs. Mediation analyses revealed that smaller preparatory responses mediated the effect of greater disinhibition (but not apathy) in FTLD but not in Alzheimer’s disease. Findings from both structural neuroimaging and functional connectivity analyses suggest that preparatory responses may be served by a neural circuit involving the vmPFC and the SN. Smaller gray matter volumes and lower functional connectivity within this circuit (particularly between the AI and ACC) were both associated with smaller preparatory responses. We also examined orienting responses but did not find any group differences, mediation effects or neural correlates.

### Diminished preparatory physiological responses in FTLD

#### Diagnostic group differences

Supporting our first hypothesis, FTLD as a group was associated with an impairment in preparatory responses, with findings being most significant for bvFTD and PSP groups. Our hypothesis that all FTLD syndromes would demonstrate impairment was not supported, although preparatory responses in svPPA and CBS were in the hypothesized direction. These physiological findings parallel recent studies reporting different degrees of behavioural changes in different FTLD syndromes. Importantly, across studies, bvFTD is typically found to show the greatest impairment, followed by svPPA, PSP or CBS; nfvPPA typically shows the least impairment compared to the other FTLD syndromes.^6,44–46^ Taken together, these findings expand upon the FTLD literature by providing further evidence of a *spectrum* of impairment in physiological and behavioural functioning across FTLD syndromes.^6,44,45^

Supporting our second hypothesis, preparatory response impairments mediated the effect of greater disinhibition in FTLD as compared to Alzheimer’s disease. The activation of the automatic nervous system (ANS), and the cardiovascular system in particular supports changes in muscle activity that are critically involved in behavioural adjustments (e.g. fighting, fleeing, freezing, approaching).^17,47^ In FTLD, reduced ANS activation before a stimulus onsets may hinder the subsequent behavioural adjustments needed to address positive and negative emotional challenges. Therefore, patients may be less prepared physiologically to mount subsequent withdrawal behaviours when negative stimuli make them feel distressed,^48^ mount subsequent approach behaviours when positive stimuli make them feel pleasant or inhibit initial responses that are inappropriate to the current situation.^6,49^ Interestingly, we did not find similar mediation effects on apathy. We suspect this may be due to the smaller group differences in apathy (Cohen’s *d* = 0.47) than disinhibition (Cohen’s *d* = 0.85). In addition, the NPI apathy score only reflected the overall severity of apathetic behaviours. Future studies are needed to systematically investigate the specific aspects of apathy (e.g. loss of interest in activities versus low motivation) that are affected by impaired preparatory responses.

Our findings let us reject several alternative hypotheses concerning preparatory responses. The first alternative hypothesis was that our results simply reflected patients’ inattention or lack of orientation to the computer monitor during the instruction period. This hypothesis is unlikely because orienting responses to external stimuli are associated with a rapid increase in IBI.^12,50^ During the instruction period of our study, the comparison groups exhibited decreased IBI, indicating preparatory responses, rather than the increased IBI that would have been consistent with the orienting response. Notably, bvFTD patients did show IBI changes consistent with the orienting responses (i.e. increased IBI) during the film period. This suggests that their ability to orient remained intact. A second alternative hypothesis is that our findings may reflect a general lowering of ANS functioning associated with older age or FTLD.^51,52^ However, we did not find significant group differences in patients’ overall physiological responding (which is consistent with the literature^30,53^ indicating that physiological responding to simple stimuli remains relatively intact in early stage of FTLD). Importantly, our main findings remained robust after adjusting for individual differences in age and overall physiological responses. These findings together undercut the likelihood of these alternative hypotheses accounting for our findings.

#### Neural correlates

The neuroimaging findings support our hypothesis that preparatory responses would be influenced by a circuit that involves the vmPFC and the SN. First, we found that smaller preparatory responses were associated with smaller gray matter volumes in the bilateral vmPFC and weaker connectivity between the vmPFC and the ACC. In the current experimental context, we believe the vmPFC is involved in stimulus evaluation (based on past experiences, social norms, etc.) and generating predictions for the future, particularly during uncertainty.^2,54,55^ The vmPFC is strongly connected to the ACC—a cortical area critical for response preparation, initiation and monitoring—including controlling the ANS via activating subcortical regions such as the PAG, which is critical for the propagation and modulation of sympathetic nervous system and parasympathetic nervous system activities.^56–58^ Co-activation of the vmPFC and ACC is often found in decision-making tasks that involve anticipation with uncertainty.^59,60^ In our study, smaller vmPFC volumes may make patients less attentive to the cues indicating that a film will start soon (e.g. instructions ‘Please watch the film’). It may also impair patients’ ability to retrieve semantic knowledge or similar memories from the past (e.g. from prior trials) and compare them with the current situation to predict the salience of upcoming emotional stimuli. The loss of functional connectivity between the vmPFC and ACC may lead to the ACC receiving partial or inappropriate signals from the vmPFC, leading to reduced ANS activation that can compromise preparation for coping with the upcoming emotional stimulus.

Second, we found smaller preparatory responses were associated with smaller gray matter volumes in the bilateral AI and weaker functional connectivity between AI and ACC—above and beyond all other node pairs examined in this study. The AI receives interoceptive signals from the body—including those reflecting cardiac activity—via relays in the Thal and posterior insula.^61^ It has been argued that the AI integrates such interoceptive signals with input from other brain regions, interprets the meaning of these signals, and generates representation into conscious awareness. The outputs of AI go to the ACC for simultaneous monitoring of current responses, detection of errors and preparation for future actions including changes in the ANS.^57,62,63^ Co-activation of AI and ACC has often been noted in studies with emotional tasks.^64,65^ In our study, the AI-ACC connection may be particularly important during physiological preparation. Input from the AI (and vmPFC) may enable the ACC to compute the predicted requirements of the body (i.e. homeostatic and coping behaviour needs) relative to its current status, which in turn can activate or deactivate the ANS.^66^ In FTLD, declines in the AI structure and its connectivity to the ACC may result in partial and inaccurate interoceptive information to the ACC, leading the ACC to underestimate the amount of ANS changes needed for the body to prepare for upcoming emotional stimuli.

Third, functional connectivity analyses also revealed that weaker *overall connectivity* among nodes in the vmPFC-SN circuit was associated with smaller preparatory responses. This relationship was not found between preparatory responses and the SMN, which underscores the specific contribution of the vmPFC–SN circuit to preparatory responding. Interestingly, several nodes in the functional connectivity analyses (e.g. posterior ACC) did not emerge in our VBM analyses. In neurodegenerative diseases like FTLD, functional decline of brain tissue typically precedes permanent structural loss.^67,68^ Additionally, disproportionate progression in gray matter tissue (e.g. ACC) versus white matter tract loss (e.g. AI to ACC) may also occur, especially in CBS and PSP.^69^ Thus, differences between our structural and functional findings may reflect the pathological complexity in neurodegenerative diseases, highlighting the need for deploying a multi-imaging-method approach in research and clinical practice.

The VBM analyses also revealed that smaller preparatory responses were associated with smaller volumes in the anterior temporal lobe and dorsal striatum (i.e. caudate). The anterior temporal lobe is strongly involved in social cognition including processing social concepts.^70–72^ In our study, patients with volume loss to this region may have encountered difficulties in accessing the meanings of the social context (i.e. participating in a study of emotion and being asked to watch films). Such information may be necessary for the vmPFC to predict the salience level of the upcoming emotional stimuli. The dorsal striatum implements motor planning^73,74^; thus, volume loss to this region may impair patients’ ability to strategize the sequence of motor actions needed for the next moment and the amount of ANS changes required for these actions. Importantly, both the anterior temporal lobe and dorsal striatum have strong connections to the vmPFC to form a ‘semantic appraisal network’ (along with other brain regions such as the orbital gyrus).^33^ Therefore, our VBM findings raise the possibility that, in addition to our hypothesized vmPFC-SN circuit, other brain regions/networks might also contribute to diminished preparatory responding. Interestingly, past research has often reported the ventral striatum and Amy as being involved in the anticipation of positive and negative emotional stimuli, respectively. Nevertheless, our VBM analyses did not reveal any significant effects in these regions. While there are many factors that might account for these non-significant findings, including study design (e.g. monetary rewards versus emotional films), patient disease severity (i.e. structural declines in these regions may occur in later disease stages) and statistical thresholds, these findings also indicate that generating preparatory responses may not require evaluating the valence of an upcoming emotional stimulus.

### Orienting responses in FTLD

Interestingly, our analyses did not reveal any diagnostic group differences, mediation effects, or neural correlates for orienting responses, which is not consistent with the existing literature.^13–16^ One factor that may contribute to these disparate findings is that these previous studies typically did not include an ‘instruction period’ that preceded the stimulus. The instruction period in our study may have attenuated the magnitude of orienting responses by making the timing of the stimulus onset more predictable. In addition, in our study, orienting and preparatory responses occurred in proximity, which could have obscured effects for both responses—particularly for orienting responses, which may have overlapped with the late phase of preparatory responses. Future studies will benefit from including trials with and without the instruction period to systematically compare preparatory versus orienting responses in FTLD and their neural correlates.

### Implications

Findings of our study have several important implications. First, our findings advance clinical characterizations of emotional and physiological responding in FTLD. Importantly, behavioural disinhibition is one prominent characteristic in FTLD; it is also a diagnostic criterion for bvFTD and PSP.^22,26^ This is consistent with our findings that impairment in preparatory responses are (i) found most prominently in bvFTD and PSP and (ii) mediate the effect of greater disinhibition in FTLD than in Alzheimer’s disease. Given the strong association between cardiovascular ANS responding and somatic muscle activity,^47^ our findings suggest that impaired preparatory responses may be one source for the behavioural symptoms in FTLD. Second, contemporary neuroscience models argue the brain is a ‘predictive machine,’ which constantly integrates exteroceptive and interoceptive information from current and past events in order to make predictions about what the brain and body will need in the next moment.^66,75^ Most of these models speculate AI–ACC–vmPFC interactions are critical for making such predictions. Our neuroimaging findings provide empirical support for these models and highlight the importance of investigating whether other brain regions are also involved (e.g. dorsal striatum, anterior temporal regions) or less important in (e.g. ventral striatum, SMN) in making these predictions. Third, methodologically, prior research has typically treated responses prior to stimuli onsets as the baseline and either excluded or adjusted for these responses in data analyses. Our findings suggest that responses during this pre-stimulus period may reflect important psychological processes. While between-group differences may emerge before stimulus onset, future research may benefit from carefully evaluating the dynamic change of responses over time.

### Strength and limitations

Our study had several strengths, including: (i) examining physiological processes during a preparatory time period that have been largely overlooked; (ii) using a large sample across the full spectrum of FTLD and Alzheimer’s disease and including HC, thus enabling us to evaluate diagnostic specificity/generalizability, maximize neuroanatomical and behavioural heterogeneity and increase statistical power; (iii) utilizing both VBM and functional connectivity analyses, allowing us to examine structural and functional changes associated with diminished preparatory responses; (iv) examining physiological responding preceding a range of emotional films, which increases the generalizability of findings; and (v) testing a number of alternative hypotheses (e.g. orienting responses, SMN connectivity) and covariates (e.g. overall physiological responding, disease severity), which helped rule out the possibility that our findings simply reflected confounding influences.

Our study also had limitations: (i) we did not include a control trial in which participants were told to wait for an emotionally neutral film to start; thus, it remains undetermined whether preparatory responses only occur preceding emotional stimuli; (ii) seeing the sentence ‘say stop if you need the film stopped’ during the instruction period may have made some participants falsely believe that all films were negatively valenced. Although our additional findings (Supplementary Fig. 4) suggest that this might not affect our results, future studies without this sentence are needed to determine whether preparatory responding occurs similarly before positive and negative stimuli; (iii) our node–pair connectivity analyses only focused on the pairs driven from our hypothesized model but not those outside the model; and (iv) although our hypothesized model suggests neural processes to be sequential and directional, our analyses only tested simultaneous covariations between nodes.

## Conclusion

This is the first study to examine preparatory responses that occur prior to the onset of emotional stimuli and their neural correlates. We report (i) FTLD, particularly bvFTD and PSP, had impaired preparatory responses; (ii) impairment in preparatory responses explained greater disinhibition—an often-observed behavioural symptom in FTLD; and (iii) smaller preparatory responses were associated with smaller volumes and lower functional connectivity in a brain circuit that involves the vmPFC and SN. These findings advance our knowledge of how FTLD can negatively impact patients’ emotional and physiological responding and produce behavioural symptoms. These findings also shed light on how predictions and preparations are made in the brain to help our bodies physiologically prepare for everyday challenges and opportunities.

## Supplementary Material

fcac075_Supplementary_Data

## Abbreviations

ACC =: anterior cingulate cortex
AI =: anterior insula
Amy =: amygdala
ANS =: automatic nervous system
bvFTD =: behavioural variant frontotemporal dementia
CBS =: corticobasal syndrome
CDR-Box =: Dementia Rating Scale sum of boxes
CDR-Total =: Dementia Rating Scale total score
FTLD =: frontotemporal lobar degeneration
HC =: healthy control
Hyp =: hypothalamus
IBI =: cardiac inter-beat intervals
MMSE =: Mini-Mental State Examination
MNI =: Montreal Neurological Institute
nfvPPA =: non-fluent variant primary progressive aphasia
PAG =: periaqueductal gray
PNS =: parasympathetic automatic nervous system
PSP =: progressive supranuclear palsy
ROIs =: regions-of-interest
rs-fMRI =: resting-state functional MRI
SMN =: sensorimotor network
SN =: salience network
SNS =: sympathetic nervous system
svPPA =: semantic variant primary progressive aphasia
Thal =: thalamus
TIV =: total intracranial volume
UCB =: Berkeley Psychophysiology Laboratory at the University of California, Berkeley
UCSF =: Memory and Aging Centere at the University of California, San Francisco
VBM =: voxel-based morphometry
vmPFC =: ventromedial prefrontal cortex

## References

[1] Roberts NA , Beer JS, Werner KH, et al The impact of orbital prefrontal cortex damage on emotional activation to unanticipated and anticipated acoustic startle stimuli. Cogn Affect Behav Neurosci. 2004;4(3):307–316. 15535166 10.3758/cabn.4.3.307

[2] Damasio AR . Descartes’ error: Emotion, reason, and the human brain. Grosset/Putnam; 1994.

[3] Hiser J , Koenigs M. The multifaceted role of the ventromedial prefrontal cortex in emotion, decision making, social cognition, and psychopathology. Biol Psychiatry. 2018;83(8):638–647. 29275839 10.1016/j.biopsych.2017.10.030 PMC5862740

[4] Sturm VE , Sible IJ, Datta S, et al Resting parasympathetic dysfunction predicts prosocial helping deficits in behavioral variant frontotemporal dementia. Cortex. 2018;109:141–155. 30317048 10.1016/j.cortex.2018.09.006 PMC6261789

[5] Seeley WW , Crawford RK, Zhou J, Miller BL, Greicius MD. Neurodegenerative diseases target large-scale human brain networks. Neuron. 2009;62(1):42–52. 19376066 10.1016/j.neuron.2009.03.024 PMC2691647

[6] Lansdall CJ , Coyle-Gilchrist ITS, Jones PS, et al Apathy and impulsivity in frontotemporal lobar degeneration syndromes. Brain. 2017;140(6):1792–1807. 28486594 10.1093/brain/awx101 PMC5868210

[7] Cummings J . The neuropsychiatric inventory: Development and applications. J Geriatr Psychiatry Neurol. 2020;33(2):73–84. 32013737 10.1177/0891988719882102 PMC8505128

[8] Eckart JA , Sturm VE, Miller BL, Levenson RW. Diminished disgust reactivity in behavioral variant frontotemporal dementia. Neuropsychologia. 2012;50(5):786–790. 22285794 10.1016/j.neuropsychologia.2012.01.012 PMC3309072

[9] Perry DC , Datta S, Sturm VE, et al Reward deficits in behavioural variant frontotemporal dementia include insensitivity to negative stimuli. Brain. 2017;140(12):3346–3356. 29053832 10.1093/brain/awx259 PMC5841034

[10] Bradley MM , Lang PJ. Affective reactions to acoustic stimuli. Psychophysiology. 2000;37(2):204–215. 10731770

[11] Bradley MM , Codispoti M, Cuthbert BN, Lang PJ. Emotion and motivation I: Defensive and appetitive reactions in picture processing. Emotion. 2001;1(3):276–298. 12934687

[12] Chen K-H , Aksan N, Anderson SW, Grafft A, Chapleau MW. Habituation of parasympathetic-mediated heart rate responses to recurring acoustic startle. Front Psychol. 2014;5:1288. 25477830 10.3389/fpsyg.2014.01288 PMC4238409

[13] Joshi A , Jimenez E, Mendez MF. Pavlov’s orienting response in frontotemporal dementia. J Neuropsychiatry Clin Neurosci. 2017;29(4):351–356. 28464702 10.1176/appi.neuropsych.17020035

[14] Polich J . Updating P300: An integrative theory of P3a and P3b. Clin Neurophysiol. 2007;118(10):2128–2148. 17573239 10.1016/j.clinph.2007.04.019 PMC2715154

[15] Williams LM , Brammer MJ, Skerrett D, et al The neural correlates of orienting: An integration of fMRI and skin conductance orienting. Neuroreport. 2000;11(13):3011–3015. 11006985 10.1097/00001756-200009110-00037

[16] Buchanan SL , Powell DA. Cingulate cortex: Its role in pavlovian conditioning. J Comp Physiol Psychol. 1982;96:755–774. 7142487 10.1037/h0077925

[17] Obrist PA , Webb RA, Sutterer JR, Howard JL. The cardiac-somatic relationship: Some reformulations. Psychophysiology. 1970;6(5):569–587. 5485542 10.1111/j.1469-8986.1970.tb02246.x

[18] Berntson GG , Quigley KS, Norman GJ, Lozano DL. Cardiovascular psychophysiology. In: Berntson GG, Cacioppo JT, Tassinary LG, eds. Handbook of Psychophysiology. 4th edn. Cambridge University Press: Cambridge Handbooks in Psychology; 2016. 183–216.

[19] Cummings JL , Mega M, Gray K, Rosenberg-Thompson S, Carusi DA, Gornbein J. The neuropsychiatric inventory: Comprehensive assessment of psychopathology in dementia. Neurology. 1994;44(12):2308–2314. 7991117 10.1212/wnl.44.12.2308

[20] McKhann GM , Knopman DS, Chertkow H, et al The diagnosis of dementia due to Alzheimer’s disease: Recommendations from the national institute on aging-Alzheimer’s association workgroups on diagnostic guidelines for Alzheimer's disease. Alzheimers Dement. 2011;7(3):263–269. 21514250 10.1016/j.jalz.2011.03.005 PMC3312024

[21] Seeley WW , Menon V, Schatzberg AF, et al Dissociable intrinsic connectivity networks for salience processing and executive control. J Neurosci. 2007;27(9):2349–2356. 17329432 10.1523/JNEUROSCI.5587-06.2007 PMC2680293

[22] Rascovsky K , Hodges JR, Knopman D, et al Sensitivity of revised diagnostic criteria for the behavioural variant of frontotemporal dementia. Brain. 2011;134(9):2456–2477. 21810890 10.1093/brain/awr179 PMC3170532

[23] Gorno-Tempini ML , Hillis AE, Weintraub S, et al Classification of primary progressive aphasia and its variants. Neurology. 2011;76(11):1006–1014. 21325651 10.1212/WNL.0b013e31821103e6 PMC3059138

[24] Armstrong MJ , Litvan I, Lang AE, et al Criteria for the diagnosis of corticobasal degeneration. Neurology. 2013;80(5):496–503. 23359374 10.1212/WNL.0b013e31827f0fd1 PMC3590050

[25] Brown JA , Hua AY, Trujillo A, et al Advancing functional dysconnectivity and atrophy in progressive supranuclear palsy. Neuroimage Clin. 2017;16:564–574. 28951832 10.1016/j.nicl.2017.09.008 PMC5605489

[26] Litvan I , Agid Y, Calne D, et al Clinical research criteria for the diagnosis of progressive supranuclear palsy (Steele-Richardson-Olszewski syndrome): Report of the NINDS-SPSP international workshop. Neurology. 1996;47(1):1–9. 8710059 10.1212/wnl.47.1.1

[27] Levenson RW . Emotion elicitation with neurological patients. In: Coan JA, Allen JJB, eds. Handbook of emotion elicitation and assessment: Oxford University Press; 2007. 158–168.

[28] Sturm VE , Yokoyama JS, Eckart JA, et al Damage to left frontal regulatory circuits produces greater positive emotional reactivity in frontotemporal dementia. Cortex. 2015;64:55–67. 25461707 10.1016/j.cortex.2014.10.002 PMC4346386

[29] Verstaen A , Eckart JA, Muhtadie L, et al Insular atrophy and diminished disgust reactivity. Emotion. 2016;16(6):903–912. 27148847 10.1037/emo0000195 PMC5009015

[30] Goodkind MS , Gyurak A, McCarthy M, Miller BL, Levenson RW. Emotion regulation deficits in frontotemporal lobar degeneration and Alzheimer’s disease. Psychol Aging. 2010;25(1):30–37. 20230125 10.1037/a0018519 PMC2841311

[31] Morris JC . The clinical dementia rating (CDR): Current version and scoring rules. Neurology. 1993;43(11):2412–2414. 10.1212/wnl.43.11.2412-a 8232972

[32] Toller G , Brown J, Sollberger M, et al Individual differences in socioemotional sensitivity are an index of salience network function. Cortex. 2018;103:211–223. 29656245 10.1016/j.cortex.2018.02.012 PMC6143366

[33] Toller G , Yang WFZ, Brown JA, et al Divergent patterns of loss of interpersonal warmth in frontotemporal dementia syndromes are predicted by altered intrinsic network connectivity. Neuroimage Clin. 2019;22:101729. 30836325 10.1016/j.nicl.2019.101729 PMC6403437

[34] Braak H , Braak E. Staging of Alzheimer's disease-related neurofibrillary changes. Neurobiol Aging. 1995;16(3):271–278. 7566337 10.1016/0197-4580(95)00021-6

[35] Folstein MF , Folstein SE, McHugh PR. “Mini-mental state”: A practical method for grading the cognitive state of patients for the clinician. J Psychiatr Res. 1975;12(3):189–198. 1202204 10.1016/0022-3956(75)90026-6

[36] Gardner RC , Boxer AL, Trujillo A, et al Intrinsic connectivity network disruption in progressive supranuclear palsy. Ann Neurol. 2013;73(5):603–616. 23536287 10.1002/ana.23844 PMC3732833

[37] Lee SE , Khazenzon AM, Trujillo AJ, et al Altered network connectivity in frontotemporal dementia with C9orf72 hexanucleotide repeat expansion. Brain. 2014;137(11):3047–3060. 25273996 10.1093/brain/awu248 PMC4208465

[38] Seeley WW , Crawford R, Rascovsky K, et al Frontal paralimbic network atrophy in very mild behavioral variant frontotemporal dementia. Arch Neurol. 2008;65(2):249–255. 18268196 10.1001/archneurol.2007.38 PMC2544627

[39] Linnman C , Moulton EA, Barmettler G, Becerra L, Borsook D. Neuroimaging of the periaqueductal gray: State of the field. Neuroimage. 2012;60(1):505–522. 22197740 10.1016/j.neuroimage.2011.11.095 PMC3288184

[40] Beissner F , Meissner K, Bär KJ, Napadow V. The autonomic brain: An activation likelihood estimation meta-analysis for central processing of autonomic function. J Neurosci. 2013;33(25):10503–10511. 23785162 10.1523/JNEUROSCI.1103-13.2013 PMC3685840

[41] Hayes AF . Introduction to mediation, moderation, and conditional process analysis: a regression-based approach: Guilford Press; 2013.

[42] Hayasaka S , Nichols TE. Combining voxel intensity and cluster extent with permutation test framework. NeuroImage. 2004;23(1):54–63. 15325352 10.1016/j.neuroimage.2004.04.035

[43] Bates E , Wilson SM, Saygin AP, et al Voxel-based lesion–symptom mapping. Nat Neurosci. 2003;6:448–450. 12704393 10.1038/nn1050

[44] Coyle-Gilchrist ITS , Dick KM, Patterson K, et al Prevalence, characteristics, and survival of frontotemporal lobar degeneration syndromes. Neurology. 2016;86(18):1736–1743. 27037234 10.1212/WNL.0000000000002638 PMC4854589

[45] Murley AG , Coyle-Gilchrist I, Rouse MA, et al Redefining the multidimensional clinical phenotypes of frontotemporal lobar degeneration syndromes. Brain. 2020;143(5):1555–1571. 32438414 10.1093/brain/awaa097 PMC7241953

[46] Brown CL , Hua AY, Coster LD, et al Comparing two facets of emotion perception across multiple neurodegenerative diseases. Soc Cogn Affect Neurosci. 2020;15(5):511–522. 32363385 10.1093/scan/nsaa060 PMC7328026

[47] Levenson RW . The Autonomic nervous system and emotion. Emot Rev. 2014;6(2):100–112.

[48] Otero MC , Levenson RW. Emotion regulation via visual avoidance: Insights from neurological patients. Neuropsychologia. 2019;131:91–101. 31082398 10.1016/j.neuropsychologia.2019.05.003 PMC6650310

[49] Chen K-H , Lwi SJ, Hua AY, Haase CM, Miller BL, Levenson RW. Increased subjective experience of non-target emotions in patients with frontotemporal dementia and Alzheimer’s disease. Curr Opin Behav Sci. 2017;15:77–84. 29457053 10.1016/j.cobeha.2017.05.017 PMC5810592

[50] Smith DBD , Strawbridge PJ. The heart rate response to a brief auditory and visual stimulus. Psychophysiology. 1969;6(3):317–329. 5353382 10.1111/j.1469-8986.1969.tb02909.x

[51] Guo CC , Sturm VE, Zhou J, et al Dominant hemisphere lateralization of cortical parasympathetic control as revealed by frontotemporal dementia. Proc Natl Acad Sci U S A. 2016;113(17):E2430–E2439. 27071080 10.1073/pnas.1509184113 PMC4855566

[52] Sturm VE , Brown JA, Hua AY, et al Network architecture underlying basal autonomic outflow: Evidence from frontotemporal dementia. J Neurosci. 2018;38(42):8943–8955. 30181137 10.1523/JNEUROSCI.0347-18.2018 PMC6191520

[53] Sturm VE , Rosen HJ, Allison S, Miller BL, Levenson RW. Self-conscious emotion deficits in frontotemporal lobar degeneration. Brain. 2006;129:2508–2516. 16844714 10.1093/brain/awl145

[54] Hsu M , Bhatt M, Adolphs R, Tranel D, Camerer CF. Neural systems responding to degrees of uncertainty in human decision-making. Science. 2005;310(5754):1680–1683. 16339445 10.1126/science.1115327

[55] Ochsner KN , Silvers JA, Buhle JT. Functional imaging studies of emotion regulation: a synthetic review and evolving model of the cognitive control of emotion. Ann N Y Acad Sci. 2012;1251:E1–E24. 23025352 10.1111/j.1749-6632.2012.06751.x PMC4133790

[56] Critchley HD , Mathias CJ, Josephs O, et al Human cingulate cortex and autonomic control: Converging neuroimaging and clinical evidence. Brain. 2003;126(10):2139–2152. 12821513 10.1093/brain/awg216

[57] Medford N , Critchley HD. Conjoint activity of anterior insular and anterior cingulate cortex: Awareness and response. Brain Struct Funct. 2010;214(5):535–549. 20512367 10.1007/s00429-010-0265-x PMC2886906

[58] Raison CL . Cingulate and Insula: The pain in the brain ts not all the same. Biol Psychiatry. 2015;77(3):205–206. 25542514 10.1016/j.biopsych.2014.11.012

[59] Cohen MX , Heller AS, Ranganath C. Functional connectivity with anterior cingulate and orbitofrontal cortices during decision-making. Brain Res Cogn Brain Res. 2005;23(1):61–70. 15795134 10.1016/j.cogbrainres.2005.01.010

[60] Critchley HD , Mathias CJ, Dolan RJ. Neural activity in the human brain relating to uncertainty and arousal during anticipation. Neuron. 2001;29(2):537–545. 11239442 10.1016/s0896-6273(01)00225-2

[61] Craig AD . How do you feel now? The anterior insula and human awareness. Nat Rev Neurosci. 2009;10(1):59–70. 19096369 10.1038/nrn2555

[62] Craig ADB . Interoception and emotion. In: Barrett LF, ed. The handbook of emotion: Guilford; 2016. 215–234.

[63] Rolls ET . The cingulate cortex and limbic systems for emotion, action, and memory. Brain Struct Funct. 2019;224(9):3001–3018. 31451898 10.1007/s00429-019-01945-2 PMC6875144

[64] Taylor KS , Seminowicz DA, Davis KD. Two systems of resting state connectivity between the insula and cingulate cortex. Hum Brain Mapp. 2009;30(9):2731–2745. 19072897 10.1002/hbm.20705 PMC6871122

[65] Klumpp H , Angstadt M, Phan KL. Insula reactivity and connectivity to anterior cingulate cortex when processing threat in generalized social anxiety disorder. Biol Psychol. 2012;89(1):273–276. 22027088 10.1016/j.biopsycho.2011.10.010 PMC3260042

[66] Barrett LF , Simmons WK. Interoceptive predictions in the brain. Nat Rev Neurosci. 2015;16(7):419–429. 26016744 10.1038/nrn3950 PMC4731102

[67] Jack CR J , Knopman DS, Jagust WJ, et al Tracking pathophysiological processes in Alzheimer's disease: An updated hypothetical model of dynamic biomarkers. Lancet Neurol. 2013;12(2):207–216. 23332364 10.1016/S1474-4422(12)70291-0 PMC3622225

[68] Zhou J , Greicius MD, Gennatas ED, et al Divergent network connectivity changes in behavioural variant frontotemporal dementia and Alzheimer’s disease. Brain. 2010;133(5):1352–1367. 20410145 10.1093/brain/awq075 PMC2912696

[69] Zanigni S , Evangelisti S, Testa C, et al White matter and cortical changes in atypical parkinsonisms: A multimodal quantitative MR study. Parkinsonism Relat Disord. 2017;39:44–51. 28291592 10.1016/j.parkreldis.2017.03.001

[70] Olson IR , Plotzker A, Ezzyat Y. The Enigmatic temporal pole: A review of findings on social and emotional processing. Brain. 2007;130(7):1718–1731. 17392317 10.1093/brain/awm052

[71] Wong C , Gallate J. The function of the anterior temporal lobe: A review of the empirical evidence. Brain Res. 2012;1449:94–116. 22421014 10.1016/j.brainres.2012.02.017

[72] Olson IR , McCoy D, Klobusicky E, Ross LA. Social cognition and the anterior temporal lobes: A review and theoretical framework. Soc Cogn Affect Neurosci. 2013;8(2):123–133. 23051902 10.1093/scan/nss119 PMC3575728

[73] Albin RL , Young AB, Penney JB. The functional anatomy of basal ganglia disorders. Trends Neurosci. 1989;12(10):366–375. 2479133 10.1016/0166-2236(89)90074-x

[74] Grillner S , Hellgren J, Ménard A, Saitoh K, Wikström MA. Mechanisms for selection of basic motor programs—roles for the striatum and pallidum. Trends Neurosci 2005;28(7):364–370. 15935487 10.1016/j.tins.2005.05.004

[75] Seth AK . Interoceptive inference, emotion, and the embodied self. Trends Cogn Sci. 2013;17(11):565–573. 24126130 10.1016/j.tics.2013.09.007

