# Supplementary material for: Diminished preparatory physiological responses in frontotemporal lobar degeneration syndromes
Source: Brain Commun. 2022 Apr 4;4(2):fcac075. doi: 10.1093/braincomms/fcac075 (PMC9014451; doi:10.1093/braincomms/fcac075)
Supplement: fcac075_Supplementary_Data [file fcac075_supplementary_data.docx]

# SUPPLEMENTAL MATERIALS

## Supplemental Procedure

Data were collected in a 3 m x 6 m room. Instructions and film clips were presented on a 21-in. monitor at a distance of 1.75 m from the participant. The first film clip (amusement) was a scene from the TV sitcom I Love Lucy (1951–1957) depicting two female workers wrapping chocolate candy. The second film clip (sadness) was a scene from the movie The Champ (1979) depicting a boy crying after his father dies. The third film clip (disgust) was a scene from the TV show Fear Factor (2001 - 2006) depicting a man sucking fluids out of cow intestines and subsequently drinking the fluid. These film clips lasted between 87 seconds to 106 seconds.

## Supplemental Physiological Methods

### Data Processing

Among the 314 participants enrolled in this study, 17 were excluded from analyses because at least 25% of their data (based on the length of full trial that include the resting, instruction, and film periods combined for the film-watching task) were removed due to outliers or errors. We further excluded 12 participants who had extremely high IBI variability during the resting period (i.e., SD of IBI over the last 40 seconds of the resting period > 2 SD of the research sample; note that we did not include the first 20 seconds of the baseline because IBI during this time period may reflect the recovery from interactions with the experimenter before the first trial and answering questions about emotions experienced after each film). A total of 285 participants remained in the main data analyses.

## Supplemental Neuroimaging Methods

### Data Acquisition

176 MRIs (79%) were acquired on a 3T Siemens (Siemens, Iselin, NJ) TIM Trio scanner equipped with a 12-channel head coil located at the UCSF Neuroscience Imaging Center using a volumetric MPRAGE sequence (160 sagittal slices; slice thickness, 1.0 mm; FOV, 256×230mm; matrix, 256×230; voxel size, 1.0×1.0×1.0mm; TR, 2,300 ms; TE, 2.98 ms; flip angle, 9°). 37 MRIs (17%) were acquired on a 4T Bruker MedSpec system at the San Francisco Veterans Administration Hospital with an 8-channel head coil controlled by a Siemens Trio console, using an MPRAGE sequence (192 sagittal slices; slice thickness, 1 mm; FOV, 256×224 mm; matrix, 256×224; voxel size, 1.0×1.0×1.0mm; TR, 2,840 ms; TE, 3 ms; flip angle, 7°). 9 MRIs (4%) were acquired on a 1.5T Siemens Magnetom VISION system (Siemens, Iselin, NJ) at the San Francisco Veterans Administration Hospital, equipped with a standard quadrature head coil, using a magnetization prepared rapid gradient echo (MPRAGE) sequence (164 coronal slices; slice thickness, 1.5 mm; field of view [FOV], 256×256mm; matrix, 256×256; voxel size, 1.0×1.5×1.0mm; repetition time [TR], 10 ms; echo time [TE], 4 ms; flip angle, 15°).

Note that there were no diagnostic differences (six patient diagnostic groups) in the proportion of MRI scans acquired through the three different scanners (*X*^2^ (12, 222) = 12.03, *P* = 0.44); also see Supplemental Table S3. Although neuroimaging analyses that include images collected across different types of scanners have robust effects and are unlikely to cause artifacts at strict statistical thresholds^1^, we included two variables for scanner types (dummy coded 1 for the scanner of interest or 0 for the remaining scanners) as covariates in all VBM analyses to account for different scanner types used for data collection.

In a subsample of 117 participants scanned on the 3 T Siemens scanner at the UCSF Neuroscience Imaging Center, task-free functional MRI images were obtained over 8 minutes on the same scanner. During data acquisition, participants were instructed to relax with their eyes closed, using a T2*-weighted gradient echo planar imaging sequence (2000 ms repetition time; 27 ms echo time; 80° flip angle; 230 × 230 mm^2^ field of view; 2.5 mm^2^ inplane voxel size; 92×92 matrix size). The sequence was acquired with an online gradient adjustment to compensate for head motion.

### Structural MRI Data Preprocessing

For structural MRI data, we utilized statistical parametric mapping version 12 (SPM12) default parameters (http://www.fil.ion.ucl.ac.uk/spm/software/spm12/) for preprocessing with the light clean-up procedure in the morphological filtering step. We then corrected structural T1 images for bias field and segmented images into gray matter, white matter, and cerebrospinal fluid, and spatially normalized into Montreal Neurological Institute (MNI) space^2^. We used default tissue probability priors (voxel size, 2.0 × 2.0 × 2.0 mm) of the International Consortium for Brain Mapping. Segmented images were then visually inspected for adequate gray matter segmentation. Smoothing was then performed on these images with an 8mm full-width at half-maximum Gaussian kernel.

### Functional MRI Data Preprocessing

Rs-fMRI data were analyzed also using SPM12. After discarding the first 5 volumes to allow for magnetic field stabilization, functional images were spatially realigned, unwarped (reduction of artifacts due to movement-by-deformation interactions), co-registered to each subject's structural T1-weighted image, normalized to the MNI T1 template, re-sampled at a voxel size of 2mm^3^, and smoothed with a 6mm full-width at half maximum Gaussian kernel. To reduce the effect of low frequency drift and high-frequency noise (Lowe et al., 1998), a low pass band filter ranging between 0.0083 and 0.15 was applied. Because head motion can induce systematic but spurious correlations particularly in older and clinical populations (Power et al., 2012), all 117 participants fulfilled all of the following criteria: translational movement ≤ 3 mm, rotational movement ≤ 3°, maximum displacement ≤ 3 mm, and spikes (=max displacement ˃ 1 mm) occurring in < 10% of the 235 volumes. Mean root-mean-square of volume-to-volume changes in translational (in mm) and rotational (mean Euler angle) movement was calculated because these metrics can be associated with ICN strength (Van Dijk et al., 2012). GLMs showed no statistical differences in translational and rotational movements between diagnostic groups (Supplemental Table S3).

For node-pair intrinsically connectivity analysis^3, 4^, each participants’ pairwise correlation coefficients were calculated between a set of cortical and subcortical regions-of-interest (ROIs), including the mPFC (±10, 11, -9), ACC (±2, 10, 40), amygdala (±20, -8, -12), hypothalamus (±4, -6, 10), PAG (dorsolateral: ±2, -32, -5; lateral: ±4, -31, -8; ventrolateral : ±3, -32, -12), thalamus (±4, -16, 8), and AI (±42, 17, -10). MARSBAR was used to create spherical ROIs centered on MNI coordinates. The MNI coordinates for the AI nodes were selected based on Seeley et al.^5^. MNI for other nodes selected based on two recent neuroimaging meta-analyses^6, 7^. Four mm spherical ROIs were centered on the peak MNI coordinates of the ventral vmPFC, AI, ACC, thalamus, and amygdala. To avoid an overlap of the ROIs centered on the right and left hypothalamus and on the PAG subregions, a ROI size of 3mm was chosen for the hypothalamus, and a ROI size of 2mm for the different PAG subregions.

A CSF mask in the central portion of the lateral ventricles and a white matter (WM) mask based on the highest probability in the FMRIB Software Library (FSL) tissue probability mask were used to extract mean CSF and WM time series. Each ROI's mean voxel-wise BOLD signal time series was used to calculate correlations with all other node-pairs, controlling for CSF, white matter, and motion regressors as described above^8^. To test our hypothesized neural circuit for preparatory physiological responses, we calculated regional summary scores by averaging each participant's correlation coefficients within each pair of nodes below: (a) vmPFC and ACC (e.g., we averaged correlation coefficients between right vmPFC to right ACC, right vmPFC to left ACC, left vmPFC to right ACC, and left vmPFC to left ACC), (b) ACC and all subcortical regions (amygdala, hypothalamus, PAG) critical for physiological activation, (c) thalamus and AI, and (d) AI and ACC, and (e) AI and vmPFC, which resulted in four correlation coefficients for each participant. Finally, for each participant we averaged these five correlation coefficients to obtain an overall index of functional connectivity for our hypothesized circuit.

To ensure our connectivity findings were specific to our ROIs or the circuit that we hypothesized, rather than reflecting a general decline in functional connectivity across other regions of the brain, we included a “control” intrinsic connected networks (ICN), the sensorimotor network (SMN). Consistent with previous studies^9, 10^, ROI-based ICN analysis was applied to identify the SMN. The MARSBAR toolbox for SPM (Brett, Anton, Valabreque, & Poline, 2002) was used to create 4 mm radius spheres centered on the right precentral gyrus (28, -16, 66), which is the hub region of the SMN according to previous evidence from healthy participants^11^. MARSBAR was also used to extract the average blood oxygen level-dependent (BOLD) signal time series of all voxels at each of the 235 volumes within right precentral gyrus (see supplementary material and methods for details). The average BOLD signal time series was then used as covariate of interest in a whole brain regression model to derive each participant's SMN t-map. Controlling for the same CSF, white matter, and motion regressors as described above, mean ICN connectivity was calculated separately for each participant's SMN t-map by computing the mean beta value across all voxels within an ICN specific mask that was height and extent thresholded at *P*_FWE_ < 0.001. The ICN's specific mask was created from an independent sample of healthy older participants (n = 30). The mask was derived by the same ROI-based ICN approach as described above, with the exception that it was created by combining the ROI-based maps seeded in the right and left hemisphere to ensure full bi-hemispheric coverage.

## Supplemental Neuroimaging Data Analysis

### Permutation analysis

Permutation analysis is a resampling approach for significance testing through which a test statistic is compared with the null distribution derived from the present study’s data set and is an accurate representation of Type 1 error at *P* < 0.05 across the entire mask. The combined peak and extent thresholds were used to determine the one-tailed *T*-threshold for multiple comparisons correction at *P*_FWE_ < 0.05. This approach has been used in similar research in this patient population^12-14^. Images were overlaid with mricron on an MNI average brain based on the gray matter templates used for preprocessing.

## Supplemental Tables

**Supplemental Table S1.** Determining the covariate variables for the mediation, VBM, and functional connectivity analyses.

**Covariates for mediation analyses (conducted based on participants with NPI apathy and disinhibition scores available)**

|  | **Preparatory physiological responses** | |  |  |
| --- | --- | --- | --- | --- |
|  | **(*n* = 247)** | |  |  |
|  | ***r*** | ***P*** |  | Note |
| **Age** | -0.13 | 0.059 |  |  |
| **Gender** | -0.003 | 0.960 |  |  |
| **Handedness** | 0.01 | 0.905 |  |  |
| **Education** | 0.01 | 0.890 |  |  |
| **Dementia severity** | -0.09 | 0.201 |  |  |
| **Cognitive functioning** | 0.04 | 0.590 |  |  |
| **Overall physiological responding** | **0.17** | **0.012** |  | Included as covariate in mediation analyses |

**Covariates for VBM analyses (conducted based on participants with structural MRI data available)**

|  | **Preparatory physiological responses** | |  |  |
| --- | --- | --- | --- | --- |
|  | **(*n* = 222)** | |  |  |
|  | ***r*** | ***p*** |  | Note |
| **Age** | -0.12 | 0.067 |  |  |
| **Gender** | 0.02 | 0.720 |  |  |
| **Handedness** | 0.00 | 0.994 |  |  |
| **Education** | 0.03 | 0.713 |  |  |
| **Dementia severity** | -0.13 | 0.058 |  | Included as covariate in VBM analyses* |
| **Cognitive functioning** | 0.04 | 0.599 |  |  |
| **Overall physiological responding** | **0.18** | **0.007** |  | Included as covariate in VBM analyses |

*Note.* Although the association between disease severity (indexed by CDR-Box) and preparatory physiological responses was only significantly trending, we still included it as a covariate in the VBM analyses because it is typically positively correlated with the severity of neurodegeneration^15^.

**Covariates for functional connectivity analyses (conducted based on participants with rs-fMRI data available)**

|  | **Preparatory physiological responses** | |  |  |
| --- | --- | --- | --- | --- |
|  | **(*n* = 117)** | |  |  |
|  | ***r*** | ***P*** |  | Note |
| **Age** | **-0.27** | **0.003** |  | Included as covariate in functional connectivity analyses |
| **Gender** | -0.01 | 0.910 |  |  |
| **Handedness** | 0.00 | 0.976 |  |  |
| **Education** | 0.01 | 0.942 |  |  |
| **Dementia severity** | 0.03 | 0.737 |  |  |
| **Cognitive functioning** | -0.03 | 0.726 |  |  |
| **Overall physiological responding** | **0.32** | **< 0.001** |  | Included as covariate in functional connectivity analyses |

**Supplemental Table S2.** Correlations between preparatory/orienting responses and functional connectivity (including our hypothesized vmPFC-SN circuit (overall and node-pair) and a control SMN network). Top: Raw scores. Bottom: Adjusted scores.

**Raw Score**

|  | **Preparatory**  **Responses** | |  | **Orienting**  **Responses** | |
| --- | --- | --- | --- | --- | --- |
|  | ***r*** | ***P*** |  | ***r*** | ***P*** |
| vmPFC-SN | **0.35** | **< 0.001** |  | 0.01 | 0.886 |
| vmPFC-ACC | **0.24** | **0.009** |  | -0.02 | 0.848 |
| *ACC-Amy/Hyp/PAG* | **0.28** | **0.002** |  | -0.01 | 0.939 |
| *Thal-AI* | **0.26** | **0.005** |  | 0.05 | 0.620 |
| *AI-ACC* | **0.29** | **0.001** |  | 0.01 | 0.884 |
| *AI-vmPFC* | **0.19** | **0.045** |  | 0.01 | 0.938 |
| SMN | 0.10 | 0.282 |  | -0.10 | 0.274 |

*Note*. Italic font indicates node-pair connectivity within the vmPFC-SN network. Bolded font indicates significant effects at the threshold of *P* < 0.05. SMN = sensorimotor network; SN = salience network; ACC = anterior cingulate cortex; AI = anterior insula; Amy = amygdala; Hyp = hypothalamus; PAG = periaqueductal gray; Thal = thalamus; vmPFC = ventromedial prefrontal cortex.

**Adjusted Score**

|  | **Preparatory**  **Responses** | |  | **Orienting**  **Responses** | |
| --- | --- | --- | --- | --- | --- |
|  | ***r*** | ***P*** |  | ***r*** | ***P*** |
| vmPFC-SN | **0.30** | **0.001** |  | 0.02 | 0.868 |
| vmPFC-ACC | **0.22** | **0.022** |  | -0.02 | 0.806 |
| *ACC-Amy/Hyp/PAG* | **0.21** | **0.032** |  | 0.05 | 0.639 |
| *Thal-AI* | 0.16 | 0.093 |  | 0.06 | 0.510 |
| *AI-ACC* | **0.29** | **0.002** |  | 0.04 | 0.694 |
| *AI-vmPFC* | 0.17 | 0.070 |  | -0.07 | 0.470 |
| SMN | 0.05 | 0.576 |  | -0.07 | 0.488 |

*Note*. Analyses adjusted for diagnostic groups, age, and overall physiological responding. Italic font indicates node-pair connectivity within the vmPFC-SN network. Bolded font indicates significant effects at the threshold of *P* < 0.05. SMN = sensorimotor network; SN = salience network; ACC = anterior cingulate cortex; AI = anterior insula; Amy = amygdala; Hyp = hypothalamus; PAG = periaqueductal gray; Thal = thalamus; vmPFC = ventromedial prefrontal cortex.

**Supplemental Table S3.** *Chi-squared* tests and one-way ANOVAs did not reveal any diagnostic differences for proportion of MRI scans acquired through the three different scanners nor translational and rotational movements during rs-fMRI data acquisition.

|  |  |  | **FTLD syndromes** | | | | |  | **Comparisons** | |  |  |  |
| --- | --- | --- | --- | --- | --- | --- | --- | --- | --- | --- | --- | --- | --- |
|  | **Total** |  | **bvFTD** | **svPPA** | **nfvPPA** | **PSP** | **CBS** |  | **AD** | **HC** |  | ***F/X^2^*** | ***P*** |
| ***MRI Scanner*** |  |  |  |  |  |  |  |  |  |  |  | 12.03 | 0.44 |
| NIC 3T | **176** |  | 31 | 23 | 26 | 21 | 27 |  | 31 | 17 |  |  |  |
| SFVA 1.5T | **9** |  | 1 | 2 | 0 | 2 | 1 |  | 2 | 1 |  |  |  |
| SFVA 4T | **37** |  | 11 | 5 | 1 | 5 | 3 |  | 10 | 2 |  |  |  |
| ***Movements during fMRI*** |  |  |  |  |  |  |  |  |  |  |  |  |  |
| Translational |  |  | 0.89  (0.11) | 0.76  (0.12) | 0.88  (0.10) | 0.82  (0.11) | 0.11  (0.09) |  | 0.87  (0.10) | 0.81  (0.15) |  | 0.91 | 0.49 |
| Rotational |  |  | 0.64  (0.11) | 0.76 (0.13) | 0.56  (0.11) | 0.65  (0.12) | 0.72  (0.10) |  | 0.75  (0.11) | 0.43  (0.17) |  | 0.72 | 0.63 |

*Note*. bvFTD = behavioral variant frontotemporal dementia; svPPA = semantic variant primary progressive aphasia; nfvPPA = non-fluent variant primary progressive aphasia; CBS = corticobasal syndrome; PSP = progressive supranuclear palsy; AD = Alzheimer’s disease; HC = healthy control.

## Supplemental Figures

##
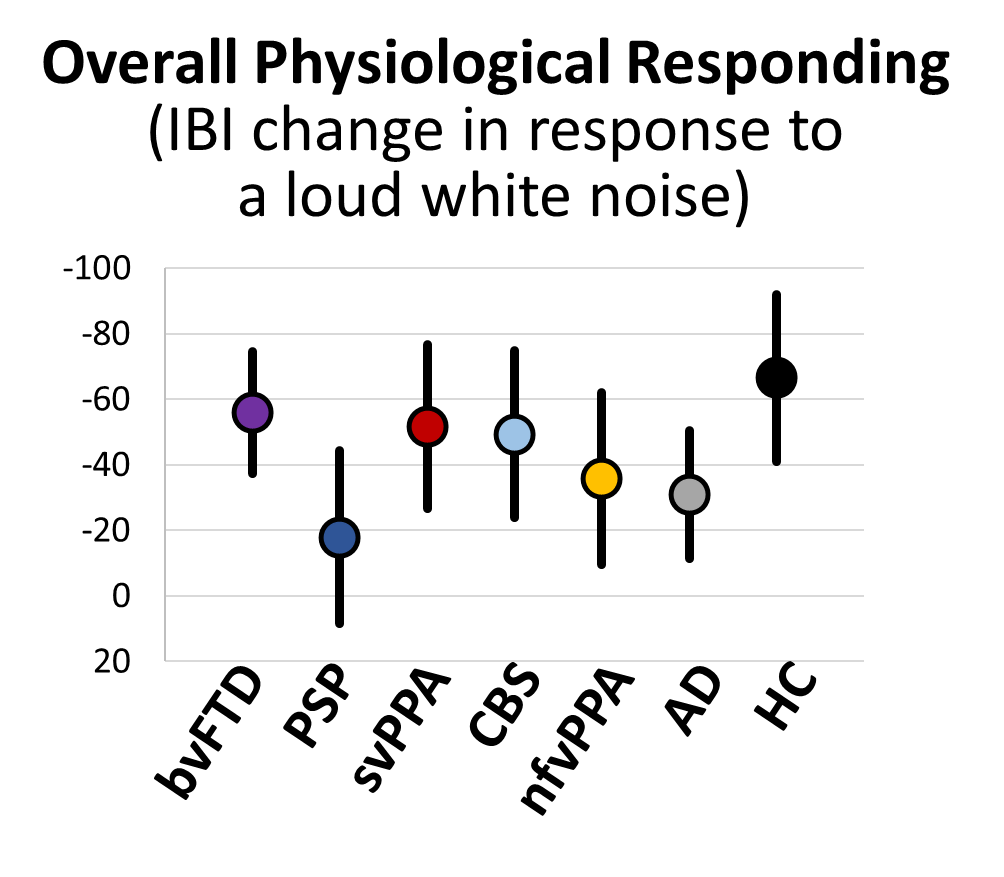


**Supplemental Fig. S1.** Averaged IBI change to a loud white noise by diagnostic groups, which was measured as a proxy for overall physiological responding. An ANOVA analyses did not reveal any significant differences between diagnostic groups (*F* = 1.85, *P* = 0.09; also see Table 1). bvFTD = behavioral variant frontotemporal dementia; svPPA = semantic variant primary progressive aphasia; nfvPPA = non-fluent variant primary progressive aphasia; CBS = corticobasal syndrome; PSP = progressive supranuclear palsy; AD = Alzheimer’s disease; HC = healthy control.


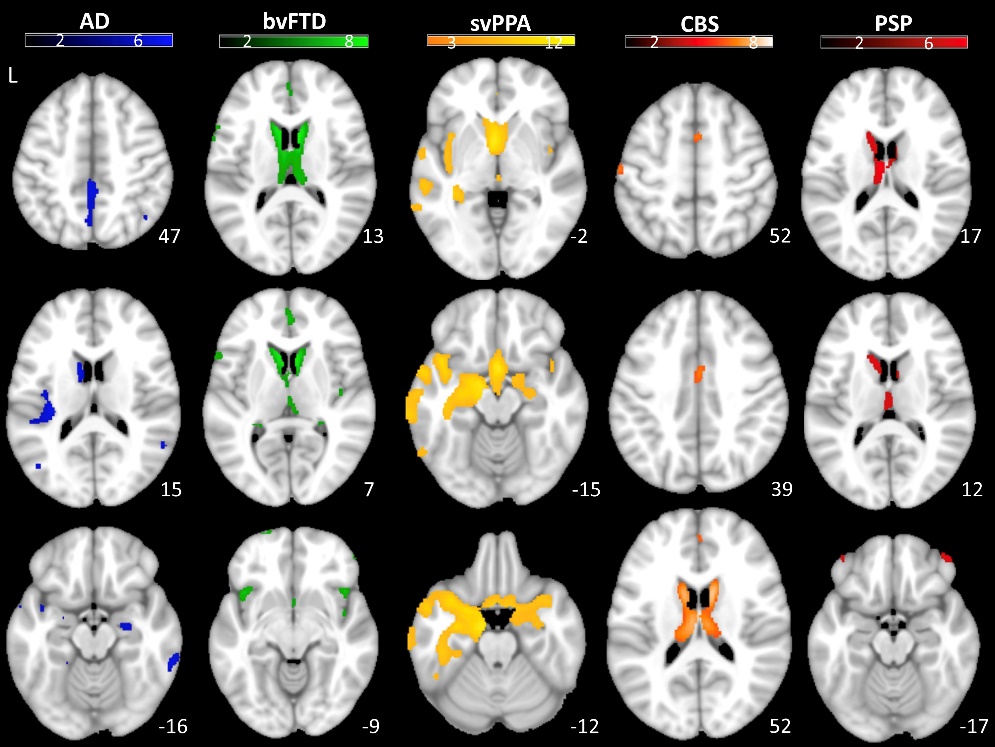


**Supplemental Fig. S2.** Distribution of neurodegeneration. Color bars represent *T*-scores for regions with smaller gray matter volume in patient groups compared to HCs after adjusting for age, sex, scanner type, and total intracranial volume (*P*_FWE_ < 0.05). Results are overlaid on an MNI template brain. The nfvPPA group did not have areas of significant volume loss compared to controls. As expected, the AD group had smaller volumes in the precuneus, hippocampus, and posterior temporal regions; the bvFTD group had smaller volumes in medial frontal, cingulate, insula, and striatum regions; the svPPA group had smaller volumes in predominantly left anterior temporal, insula, amygdala, and striatum regions; the CBS group had smaller volumes in supplementary motor area, medial frontal, cingulate, and striatum regions; and the PSP group had smaller volumes in the orbitofrontal, caudate, and thalamus regions^9, 17^. Presumably due to being in earlier stages of the disease (i.e., as indexed by lower CDR scores), the nfvPPA group did not show significant volume loss as compared to HCs (*P*_FWE_ > 0.05). AD = Alzheimer’s disease; bvFTD = behavioral variant frontotemporal dementia; svPPA = semantic variant primary progressive aphasia; CBS = corticobasal syndrome; PSP = progressive supranuclear palsy; HC = healthy control; nfvPPA = non-fluent variant primary progressive aphasia.

**
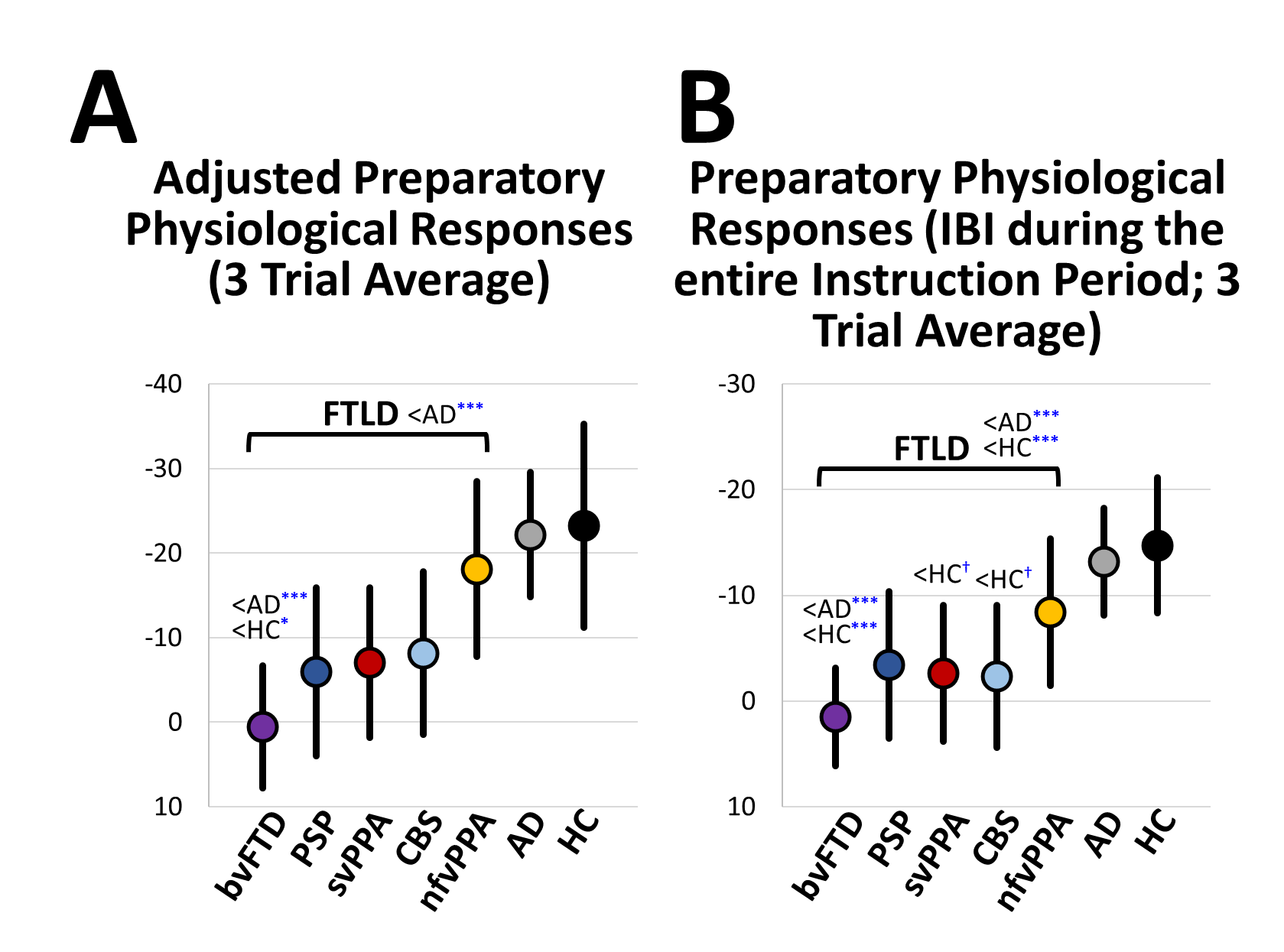
**

**Supplemental Fig. S3**. (**S3A**) Three-trial average of preparatory physiological responses adjusted for age, gender, dementia severity (CDR-Box), and cognitive functioning (MMSE), by diagnostic groups. *Mean* ± 95% confidence intervals. To ensure these effects were not driven by demographic or functional differences between diagnostic groups (Table 1), we repeated the main ANOVA analyses with an ANCOVA, including variables that significantly differed between groups. Results were very similar to the primary data analyses: when comparing FTLD, AD, and HC, *F*(2, 264) = 7.99, *P* < 0.001; FTLD < AD, *P* < 0.001; FTLD < HC, *P* = 0.125; when comparing FTLD syndromes with AD or HC, *F*(6, 260) = 3.95, *P* < 0.001; bvFTD < AD, *P* < 0.001; bvFTD < HC, *P* < 0.05. (**S3B**) In our study, preparatory physiological responses were quantified as the change in the averaged IBI of the last three seconds of the baseline period and seconds 4-6 of the instruction period (Fig. 2B; time windows W2 - W1). To ensure our primary findings using this approach were robust, we repeated our analyses using the change in the averaged IBI of the last three seconds of the baseline period and the entire six seconds of the instruction period. ANAOVs and *post hoc* tests revealed very similar results to the primary findings: when comparing FTLD, AD, and HC, *F*(2, 282) = 11.16, *P* < 0.001; FTLD < AD, *P* < 0.001; FTLD < HC, *P* < 0.001; when comparing FTLD syndromes with AD or HC, *F*(6, 278) = 4.69, *P* < 0.001; bvFTD < AD, *P* < 0.001; bvFTD < HC, *P* < 0.001. FTLD = frontotemporal lobar degeneration; bvFTD = behavioral variant frontotemporal dementia; svPPA = semantic variant primary progressive aphasia; nfvPPA = non-fluent variant primary progressive aphasia; CBS = corticobasal syndrome; PSP = progressive supranuclear palsy; AD = Alzheimer’s disease; HC = healthy control.  ^Ϯ^*P* < 0.10; **P* < 0.05; ***P* < 0.01; ****P* < 0.001.


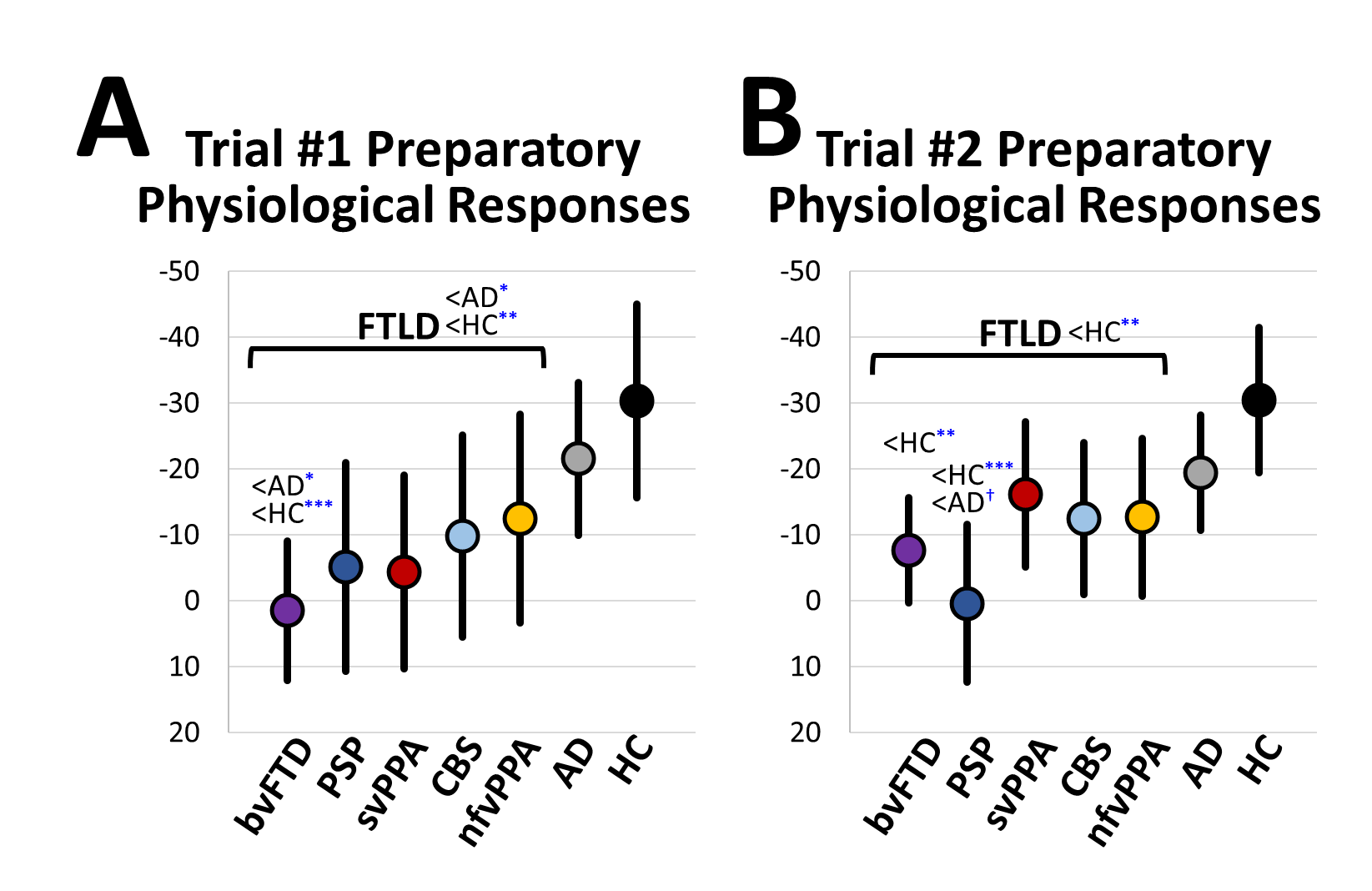


**Supplemental Fig. S4**. Preparatory physiological responses raw score for first (amusement film) and second (sadness film) trials by diagnostic groups. *Mean* ± 95% confidence intervals. **(S4A)** To ensure our findings were not biased by increased knowledge about the task after the first trial, we completed analyses using only preparatory physiological responses from the first trial. ANOVAs revealed very similar group effects as in the main analyses: when comparing FTLD, AD, and HC, *F*(2, 272) = 7.05, *P* = 0.001; FTLD < AD or HC (*P*s < 0.05); when comparing each FTLD syndrome against AD or HC, *F*(6, 278) = 2.79, *P* = 0.012; bvFTD < AD: *P* = 0.04; bvFTD < HC, *P* < 0.001. **(S4B)** To ensure our effects did not result from participants’ incorrect beliefs that the films would be always negative, we performed additional analyses focusing on preparatory physiological responses in the second trial only, which took place after participants watched an amusement film clip in the first trial (thus participants realized the films could also be positive). Again, ANOVAs (*F*s > 3.07, *P* < 0.006) and *post hoc* analyses revealed very similar group effects as reported in the main analyses, FTLD < HC: *P* = 0.002; bvFTD < HC: *P* = 0.006; nfvPPA < HC, *P* < 0.001; nfvPPA < AD, *P* = 0.095. FTLD = frontotemporal lobar degeneration; bvFTD = behavioral variant frontotemporal dementia; svPPA = semantic variant primary progressive aphasia; nfvPPA = non-fluent variant primary progressive aphasia; CBS = corticobasal syndrome; PSP = progressive supranuclear palsy; AD = Alzheimer’s disease; HC = healthy control. ^Ϯ^*P* < 0.10; **P* < 0.05; ***P* < 0.01; ****P* < 0.001.

**
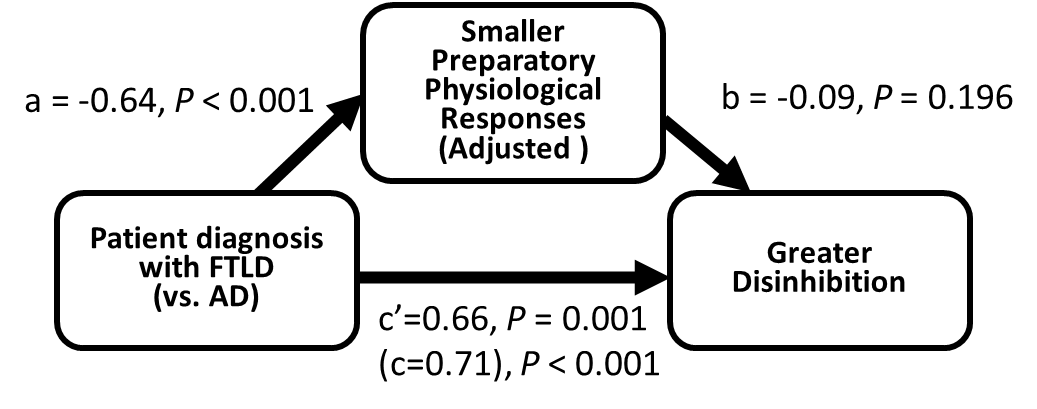
**

**Supplemental Fig. S5.** When overall physiological responding was adjusted, we observed a marginally significant effect that preparatory physiological responses mediated diagnostic group differences between FTLD and AD in disinhibition (standardized indirect effect = 0.06, 90% CI [0.0010, 0.1331], accounting for 8.15% of the total effect). FTLD = frontotemporal lobar degeneration; AD = Alzheimer’s disease.

**
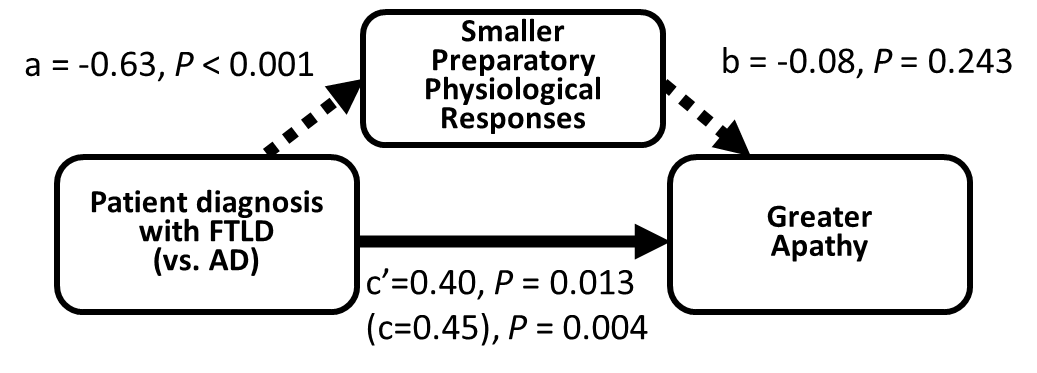
**

**Supplemental Fig. S6.** Preparatory physiological responses did not significantly mediate diagnostic group differences between FTLD and AD in apathy (standardized indirect effect = 0.0487, 95% CI [-0.0650, 0.1597], accounting for 10.74% of the total effect). FTLD = frontotemporal lobar degeneration; AD = Alzheimer’s disease.

**
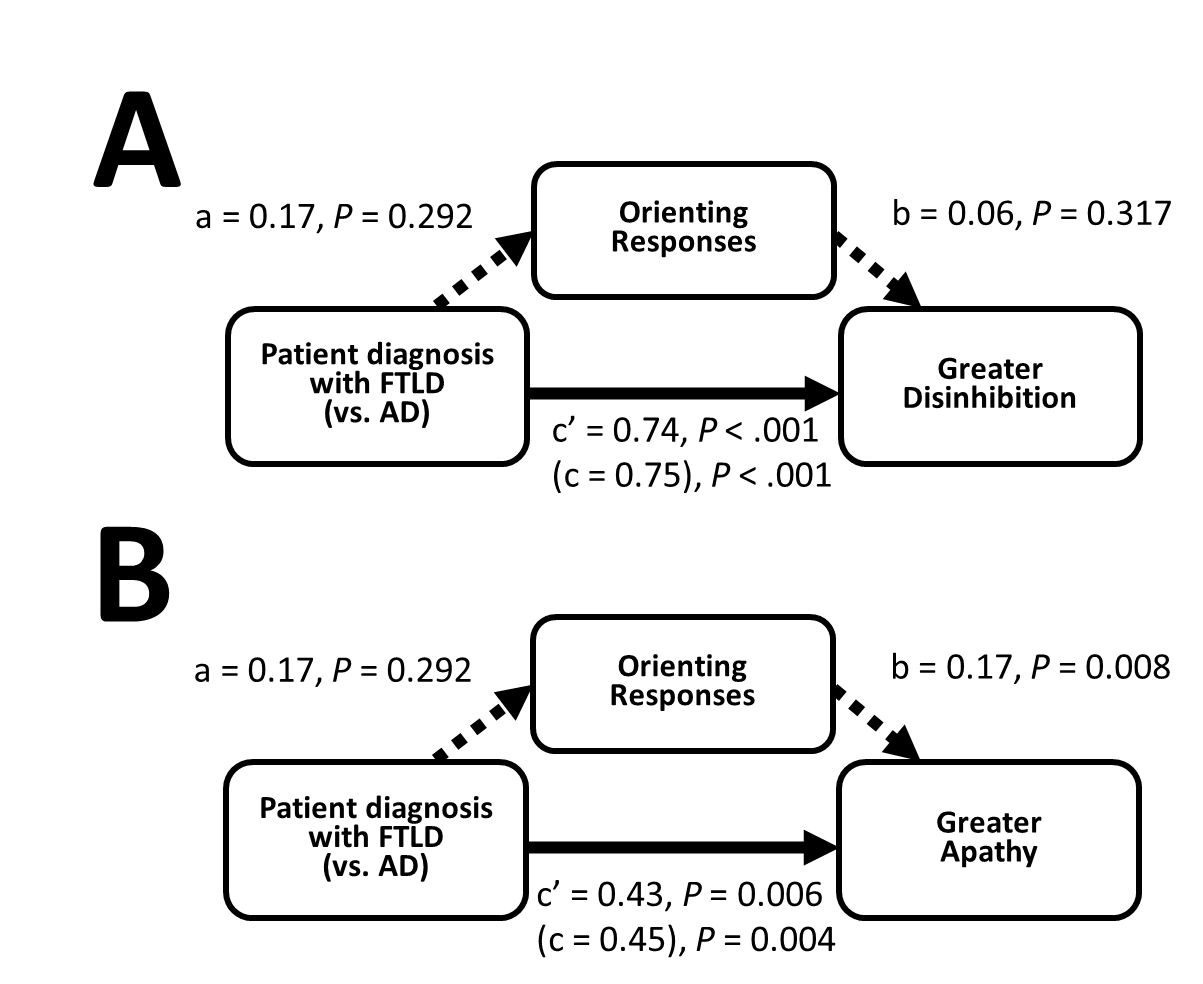
**

**Supplemental Fig. S7.** Orienting responses did not significantly mediate diagnostic group differences between FTLD and AD in **(S7A)** disinhibition (standardized indirect effect = 0.0103, 95% CI [-0.0164, 0.0766], accounting for 1.40 % of the total effect) or **(S7B)** apathy (standardized indirect effect = 0.0279, 95% CI [-0.0381, 0.1152], accounting for 6.15 % of the total effect). FTLD = frontotemporal lobar degeneration; AD = Alzheimer’s disease.

## Supplemental References

1. Abdulkadir A, Mortamet B, Vemuri P, Jack Jr CR, Krueger G, Klöppel S. Effects of hardware heterogeneity on the performance of SVM Alzheimer's disease classifier. *NeuroImage*. 10/1/ 2011;58(3):785-792. doi:<http://dx.doi.org/10.1016/j.neuroimage.2011.06.029>

2. Ashburner J, Friston KJ. Unified segmentation. *NeuroImage*. 7/1/ 2005;26(3):839-851. doi:<http://dx.doi.org/10.1016/j.neuroimage.2005.02.018>

3. Gardner RC, Boxer AL, Trujillo A, et al. Intrinsic connectivity network disruption in progressive supranuclear palsy. *Annals of Neurology*. 2013;73(5):603-616. doi:10.1002/ana.23844

4. Lee SE, Khazenzon AM, Trujillo AJ, et al. Altered network connectivity in frontotemporal dementia with C9orf72 hexanucleotide repeat expansion. *Brain*. 2014;137(11):3047-3060. doi:10.1093/brain/awu248

5. Seeley WW, Crawford R, Rascovsky K, et al. Frontal paralimbic network atrophy in very mild behavioral variant frontotemporal dementia. *Archives of Neurology*. 2008;65(2):249-255. doi:10.1001/archneurol.2007.38

6. Linnman C, Moulton EA, Barmettler G, Becerra L, Borsook D. Neuroimaging of the periaqueductal gray: State of the field. *NeuroImage*. 2012/03/01/ 2012;60(1):505-522. doi:<https://doi.org/10.1016/j.neuroimage.2011.11.095>

7. Beissner F, Meissner K, Bär KJ, Napadow V. The autonomic brain: an activation likelihood estimation meta-analysis for central processing of autonomic function. *J Neurosci*. Jun 19 2013;33(25):10503-11. doi:10.1523/jneurosci.1103-13.2013

8. Toller G, Brown J, Sollberger M, et al. Individual differences in socioemotional sensitivity are an index of salience network function. *Cortex*. 2018/06/01/ 2018;103:211-223. doi:<https://doi.org/10.1016/j.cortex.2018.02.012>

9. Seeley WW, Crawford RK, Zhou J, Miller BL, Greicius MD. Neurodegenerative diseases target large-scale human brain networks. *Neuron*. 4/16/ 2009;62(1):42-52. doi:<http://dx.doi.org/10.1016/j.neuron.2009.03.024>

10. Toller G, Yang WFZ, Brown JA, et al. Divergent patterns of loss of interpersonal warmth in frontotemporal dementia syndromes are predicted by altered intrinsic network connectivity. *NeuroImage: Clinical*. 2019/01/01/ 2019;22:101729. doi:<https://doi.org/10.1016/j.nicl.2019.101729>

11. Zielinski BA, Gennatas ED, Zhou J, Seeley WW. Network-level structural covariance in the developing brain. *Proceedings of the National Academy of Sciences*. 2010;107(42):18191-18196. doi:10.1073/pnas.1003109107

12. Sturm VE, Yokoyama JS, Seeley WW, Kramer JH, Miller BL, Rankin KP. Heightened emotional contagion in mild cognitive impairment and Alzheimer’s disease is associated with temporal lobe degeneration. *Proceedings of the National Academy of Sciences*. June 11, 2013 2013;110(24):9944-9949. doi:10.1073/pnas.1301119110

13. Yokoyama JS, Bonham LW, Sturm VE, et al. The 5-HTTLPR variant in the serotonin transporter gene modifies degeneration of brain regions important for emotion in behavioral variant frontotemporal dementia. *NeuroImage: Clinical*. 2015/01/01/ 2015;9:283-290. doi:<https://doi.org/10.1016/j.nicl.2015.07.017>

14. Sturm VE, Brown JA, Hua AY, et al. Network architecture underlying basal autonomic outflow: Evidence from frontotemporal dementia. *The Journal of Neuroscience*. 2018;doi:10.1523/jneurosci.0347-18.2018

15. Braak H, Braak E. Staging of alzheimer's disease-related neurofibrillary changes. *Neurobiology of Aging*. 5// 1995;16(3):271-278. doi:<http://dx.doi.org/10.1016/0197-4580(95)00021-6>

16. Cummings JL, Mega M, Gray K, Rosenberg-Thompson S, Carusi DA, Gornbein J. The Neuropsychiatric Inventory: comprehensive assessment of psychopathology in dementia. *Neurology*. Dec 1994;44(12):2308-14. doi:10.1212/wnl.44.12.2308

17. Brown JA, Hua AY, Trujillo A, et al. Advancing functional dysconnectivity and atrophy in progressive supranuclear palsy. *NeuroImage: Clinical*. 2017/01/01/ 2017;16:564-574. doi:<https://doi.org/10.1016/j.nicl.2017.09.008>
